# Synthesis and physicochemical characterization of acyl myricetins as potential anti-neuroexocytotic agents

**DOI:** 10.1038/s41598-023-32361-6

**Published:** 2023-03-29

**Authors:** Sora Cho, Byoungjae Kong, Younghun Jung, Jonghyeok Shin, Myungseo Park, Woo-Jae Chung, Choongjin Ban, Dae-Hyuk Kweon

**Affiliations:** 1 Interdisciplinary Program in BioCosmetics, Sungkyunkwan University, 2066 Seoburo, Suwon, Gyeonggi 16419 Republic of Korea; 2 Center for Nanomedicine, Wilmer Eye Institute, Johns Hopkins University School of Medicine, Baltimore, MD 21231 USA; 3 Department of Ophthalmology, Johns Hopkins University School of Medicine, Baltimore, MD 21231 USA; 4 Wallace H. Coulter Department of Biomedical Engineering, Emory University and Georgia Institute of Technology, 313 Ferst Drive, Atlanta, GA 30332 USA; 5 Synthetic Biology Research Center, Korea Research Institute of Bioscience and Biotechnology (KRIBB), Daejeon, 34141 Republic of Korea; 6 Environmental Health Sciences, School of Public Health, University of Minnesota, Saint Paul, MN 55108 USA; 7 Department of Integrative Biotechnology, Sungkyunkwan University, 2066 Seoburo, Suwon, Gyeonggi 16419 Republic of Korea; 8 Institute of Biomolecule Control, Sungkyunkwan University, 2066 Seoburo, Suwon, Gyeonggi 16419 Republic of Korea; 9 Biologics Research Center, Sungkyunkwan University, 2066 Seoburo, Suwon, Gyeonggi 16419 Republic of Korea; 10 Department of Environmental Horticulture, University of Seoul, 163 Seoulsiripdaero, Dongdaemun-Gu, Seoul, 02504 Republic of Korea

**Keywords:** Chemical modification, Biochemical assays, Liquid chromatography, Cell culture, ELISA, Mass spectrometry, Biophysical chemistry, Chemical modification, Mechanism of action, Small molecules, Synaptic vesicle exocytosis, Molecular medicine, Neurology, Biomaterials

## Abstract

Acyl myricetins (monopropionyl-, dipropionyl-, and monooctanoyl-myricetin, termed as MP_1_, MP_2_, and MO_1_, respectively) were synthesized through enzymatic or non-enzymatic esterification reaction of myricetin aglycone. Structure study indicated the hydroxyl group at C4′ in B-ring was highly susceptible to acylation. Over its parental myricetin, acylated compounds showed enhanced lipophilicity (from 7.4- to 26.3-fold) and oxidative stability (from 1.9- to 3.1-fold) on the basis of log*P* and decay rate, respectively. MO_1_, presenting the physicochemical superiority compared to the others, provided lowest EC_50_ value of 2.51 μM on inhibition of neutrotransmitter release and CC_50_ value of 59.0 μM, leading to widest therapeutic window. All myricetin esters did not show any irritation toxicity when assessed with a chicken embryo assay. This study describes information on acylation of myricetin that has not yet been explored, and suggests that MO_1_ has membrane fusion-arresting and anti-neuroexocytotic potential for industrial application due to its enhanced biological properties.

## Introduction

Flavonoids are aromatic secondary metabolites naturally synthesized by plants and fungi. They have been reported to possess health beneficial effects such as anti-oxidant, anti-mutagenic, anti-bacterial, anti-angiogenic, anti-inflammatory, anti-allergic, and anti-cancer activities^1,2^. While flavonoids are one of the most abundant natural resources on earth, their industrial use is still limited to foods because of poor physicochemical properties. Flavonoids are unstable in aqueous conditions, particularly at higher temperatures^3^, and show poor solubility in both oils and aqueous media^4^. Furthermore, oxidative decay of flavonoids is frequently observed during further processing and storage, thereby reducing health functionality, anti-oxidant capacity^5^, and bioavailability^6^.

Flavonoids are frequently converted to other forms to enhance their economic value in various industrial fields for foods, pharmaceuticals, cosmetics, and feedstocks^7^. The modifications include acylation to improve their solubility, to better prepare formulations, and to prolong shelf-life via chemical or enzymatic reactions^5^. The acylation of flavonoids has been examined to modulate physicochemical and biological activities by altering their solubility, stability, anti-oxidant activity, and interaction of the flavonoid esters with targeted cells^8^. Specifically, monoacylation of quercetin glucoside at primary alcohol in the glucose moiety enhances its thermal stability, light resistivity^9^, lipophilic solubility, and its activity as an anti-oxidant in vitro and in vivo^10^. Acylation is frequently performed enzymatically with flavonoid glycosides rather than with aglycones due to the regiospecific reaction with hydroxyl groups^11^. However, using aglycones for acylation is more demanding not only because the absence of sugar moieties provides better lipophilicity^12^, but also because reactive hydroxyl groups in B-ring can be masked by acyl groups^5^. Most enzymatic acylation studies for aglycone usage conducted so far employ a lipase PS from *Burkholderia cepacian*, a carboxyl esterase from *Streptomyces rochei* and *Aspergillus niger,* and lipase B from *Candida antarctica* (so called CaLB)^5,11,13^.

Myricetin, a common plant-derived flavonoid, is known to have various health benefits on the basis of its anti-oxidant^14^, anti-cancer^15,16^, neuroprotective^17^, and various other effects^18^. We also have previously shown that myricetin plays a regulatory role in the release of neurotransmitters such as acetylcholine from neuronal PC-12 cells by interfering with formation of soluble *N*-ethyl maleimide-sensitive fusion protein attachment protein receptors (SNARE) protein complex^19,20^. The mechanism study found that membrane fusion essential for neuroexocytosis is arrested by the wedge-like behavior of myricetin into the inner layer of the SNARE complex. Particularly, in vitro studies have suggested that various stages of membrane fusion, including docking, hemifusion, and pore formation, are affected by myricetin via its direct interaction with SNARE complex intermediates and with membranes^19,20^. As such, it is most likely that myricetin is useful to treat hypersecretion diseases caused by dysfunction of exocytosis, which is driven by SNARE-mediated membrane fusion. Moreover, given that botulinum neurotoxin (BoNT), the most poisonous biological drug acting by irreversibly proteolyzing the SNARE protein, was the obligate option for the treatment of hypersecretion disease, myricetin provides a promise to expand its industrial application and benefits by replacing it. However, like other flavonoids described above, the modification of myricetin is necessary to improve thermal stability and lipophilicity, which are practically required properties for industrial utilization^21^. To the best of our knowledge, little is known about the modification of aglyconic myricetin, especially of its acylation, in both enzymatic and non-enzymatic manner.

In this study, myricetin was acylated using either vinyl propionate (VP) or vinyl octanoate (VO) as an acyl donor in the presence or absence of CaLB and trimethylamine (TMA) as the enzymatic and chemical catalyst, respectively. The acylation pattern of myricetin was compared between the catalysts. The acylated products were identified using nuclear magnetic resonance (NMR) spectroscopy and mass spectrometry, and their lipophilicity and physicochemical stability were evaluated by logarithm values of the 1-octanol/water partition coefficients (log*P*) and ultraviolet/visible (UV/Vis) light absorption spectra, respectively. BoNT-like activity of acyl myricetins on neurotransmitter release was assessed using neuronal PC-12 cells. Safety of acyl myricetins were compared by evaluating cytotoxicity and *in ovo* toxicity using PC-12 cells and hen egg chorioallantoic membrane test (HET-CAM), respectively.

## Results and discussion

### Acylation of myricetin

**Figure 1:**
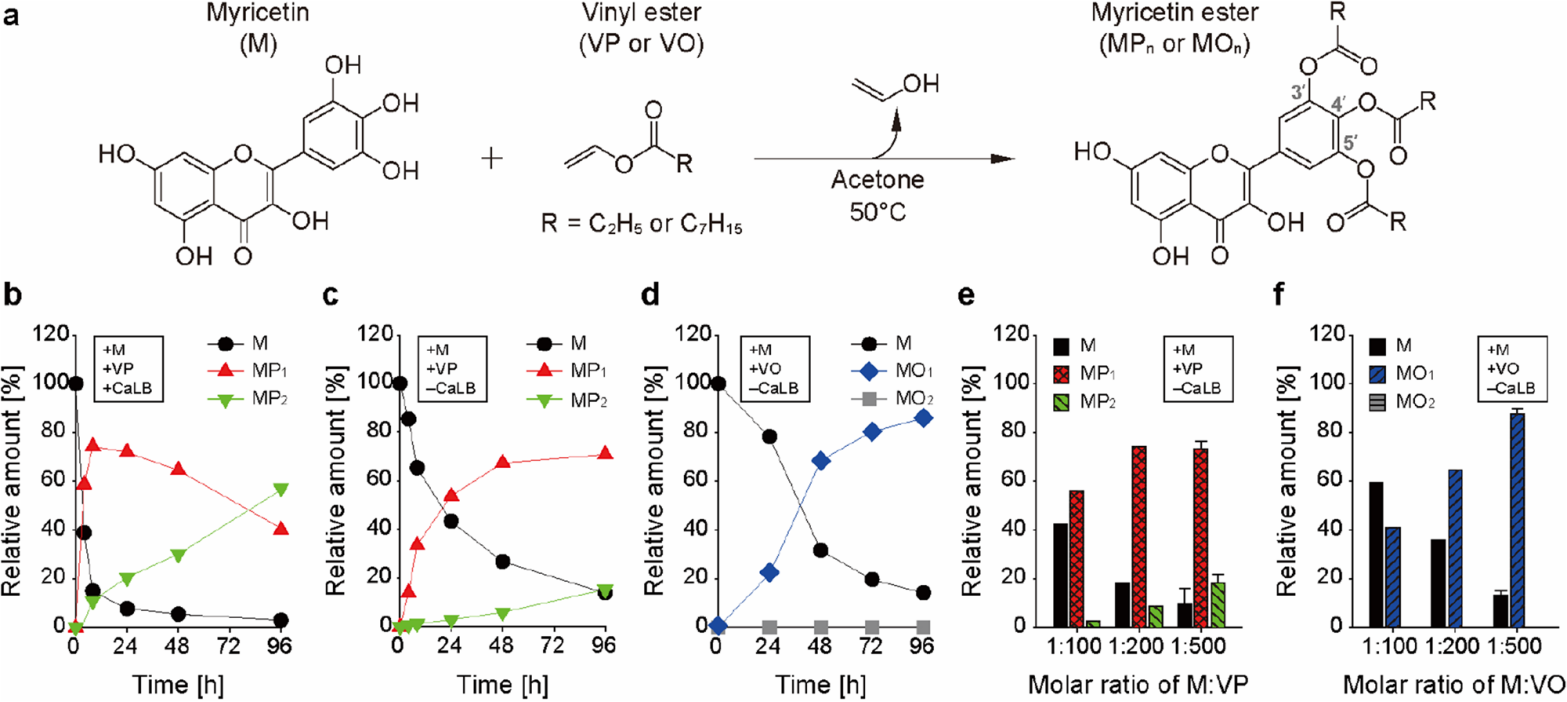
Synthesis of acyl myricetins in the presence and absence of an enzyme catalyst CaLB. (**a**) Schematic representation of acylation reaction of myricetin with vinyl ester [VP, vinyl propionate (R = C_2_H_5_); VO, vinyl octanoate (R = C_7_H_15_)], resulting in acyl myricetins. The reactions were conducted by shaking with 700 rpm at 50 °C in acetone. Three carbon positions susceptible to acylation are indicated as gray color in myricetin ester structure. (**b**–**d**) Acylation profiles of myricetin. (**b**) Myricetin (0.3 mg·mL^−1^) was acylated at a molar ratio of 1:500 with VP in the presence of 20 mg·mL^−1^ CaLB. For spontaneous acylation in the absence of CaLB, VP (**c**) or VO (**d**) as an acyl group donor was used in a reaction adopting the same conditions as in (**b**). (**e**, **f**) Effects of molar ratio of myricetin-to-vinyl ester on acylation efficiency. The relative amounts of free, monoacyl-, and diacyl-myricetin were determined after propionylation (**e**) and octanoylation (**f**) in 96-h reaction without CaLB, depending on indicated molar ratio of myricetin and vinyl ester. Reactants used for each reaction are indicated (**b**–**f**). Data are expressed as the mean ± s.d (**e**, **f**). M, myricein; MP_1_, monopropionyl-myricetin; MP_2_, dipropionyl-myricetin; MO_1_, monooctanoyl-myricetin; MO_2_, dioctanoyl-myricetin.

To synthesize acyl myricetins, we employed vinyl esters including VP and VO as acyl group donors in esterification reaction conducted in acetone at 50 °C (Fig. 1a). First, acylation of myricetin using VP acyl donor was performed in the presence of a biocatalyst CaLB under an optimized condition for flavonoid esterification (Fig. 1b)^13^. We found that almost 90% of myricetin was esterified in 8 h, during which time monopropionyl-myricetin (MP_1_) reached ~ 74%. Moreover, for overall incubation time, the ratio of dipropionyl-myricetin (MP_2_) gradually increased correspondingly with the decline of MP_1_, indicating the conversion between these two compounds. After 96 h of reaction, the final relative amounts of MP_1_ and MP_2_ was ~ 41% and ~ 56%, respectively.

Surprisingly, we observed spontaneous esterification of myricetin in the absence of CaLB enzyme in the control experiment, which was further studied by performing the reactions with myricetin using VP or VO. The propionylation profile indicated that the uncatalyzed reaction yielded ~ 70% of MP_1_ and ~ 15% of MP_2_ in 96 h, although it showed much slower rate than that of the CaLB-mediated reaction (Fig. 1c). Gradual increase in the fraction of MP_2_ was observed again in this reaction without CaLB. Spontaneous octanoylation was also found in the CaLB-lacking reaction when VO, characterized by the longer chain than VP, was utilized, resulting in 86% yield of monooctanoyl-myricetin (MO_1_) after 96-h reaction (Fig. 1d). It is notable that dioctanoyl-myricetin (MO_2_) was not synthesized at this time. We tested an effect of molar ratio of myricetin-to-vinyl esters on non-catalyzed esterification and found that lowering molar ratio caused a decrease in the degree of myricetin esterification regardless of initial concentration of myricetin, emphasizing the role of vinyl esters for successful myricetin acylation (Fig. 1e,f and Fig. S1). Collectively, it is revealed that the maximal acylation level of myricetin can be obtained in high ratio of vinyl esters under the catalytic activity of CaLB. However, considering several limitations such as enzymatic stability and high cost of a biocatalyst like CaLB for feasibility of future industrial application^22^, we sought to examine an alternative chemical catalyst to improve an efficiency of myricetin acylation.

**Figure 2:**
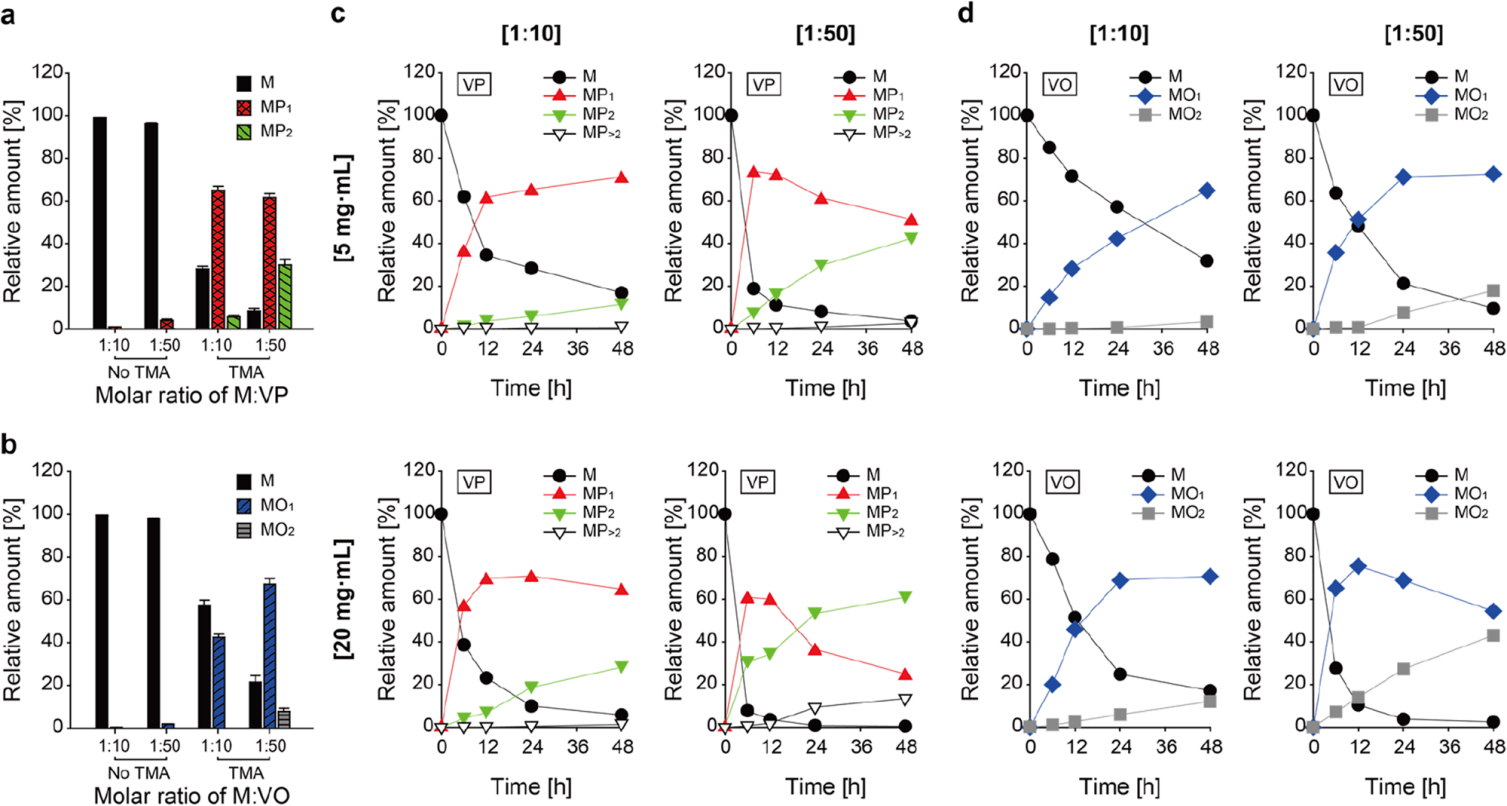
TMA-catalyzed myricetin acylation under various reaction conditions. (**a**, **b**) Investigation for effect of a chemical catalyst TMA on myricetin esterification. Acylation was performed in acetone with TMA (10 mol% of 5 mg·mL^−1^ myricetin) for 48 h, using (**a**) vinyl propionate (VP) or (**b**) vinyl octanoate (VO) as acyl donors. Data are expressed as the mean ± s.d. (**c**, **d**). Profiles of myricetin acylation where initial myricetin concentrations were 5 or 20 mg·mL^−1^, and molar ratios of myricetin-to-vinyl ester were 1:10 or 1:50. The acyl donor used for each reaction is indicated in each profile. MP_>2_, multipropionyl-myricetin.

Using TMA as a basic catalyst, the acylation of hydroxyl groups-containing polyphenols including flavonoids can be achieved as described elsewhere^23,24^. We thus adopted TMA as the catalyst of myricetin acylation reaction, where concentration of myricetin was elevated to 5 or 20 mg mL^−1^ and molar ratio of myricetin-to-acyl donors was lowered to 1:10 or 1:50. Reaction conditions with addition of TMA catalyst formed large amounts of esterified products, especially at high molar ratio of myricetin to vinyl esters (1:50), whereas non-catalyzed conditions failed to do so (Fig. 2a,b). Moreover, the most active tendency for 48-h acylation of myricetin was found in a condition using 20 mg mL^−1^ of myricetin at a myricetin:vinyl ester molar ratio of 1:50, leading to ~ 25% of MP_1_ and ~ 61% of MP_2_ for propionylation (Fig. 2c), and ~ 54% of MO_1_ and ~ 42% of MO_2_ for octanoylation (Fig. 2d), respectively. Specifically, it is notable that reaction rate of propionlyation was faster than that of octanoylation in all tested conditions. Additionally, production of multipropionated myricetins (MP_>2_) was also observed (Fig. 2c). Throughout the study on these reaction profiles, we found that myricetin esterification, showing relatively fast kinetics and subsequent resultant high productivity, benefits from increased initial myricetin concentration and acyl donor ratio. For our subsequent studies, we used the above-mentioned reaction conditions [i.e., acylation using 20 mg mL^−1^ of myricetin, catalyzed by TMA (10 mol% of myricetin) at a molar ratio of 1:50 for myricetin-to-vinyl ester in acetone at 50 °C for 48 h].

### Purification and identification of the acyl myricetins

**Figure 3:**
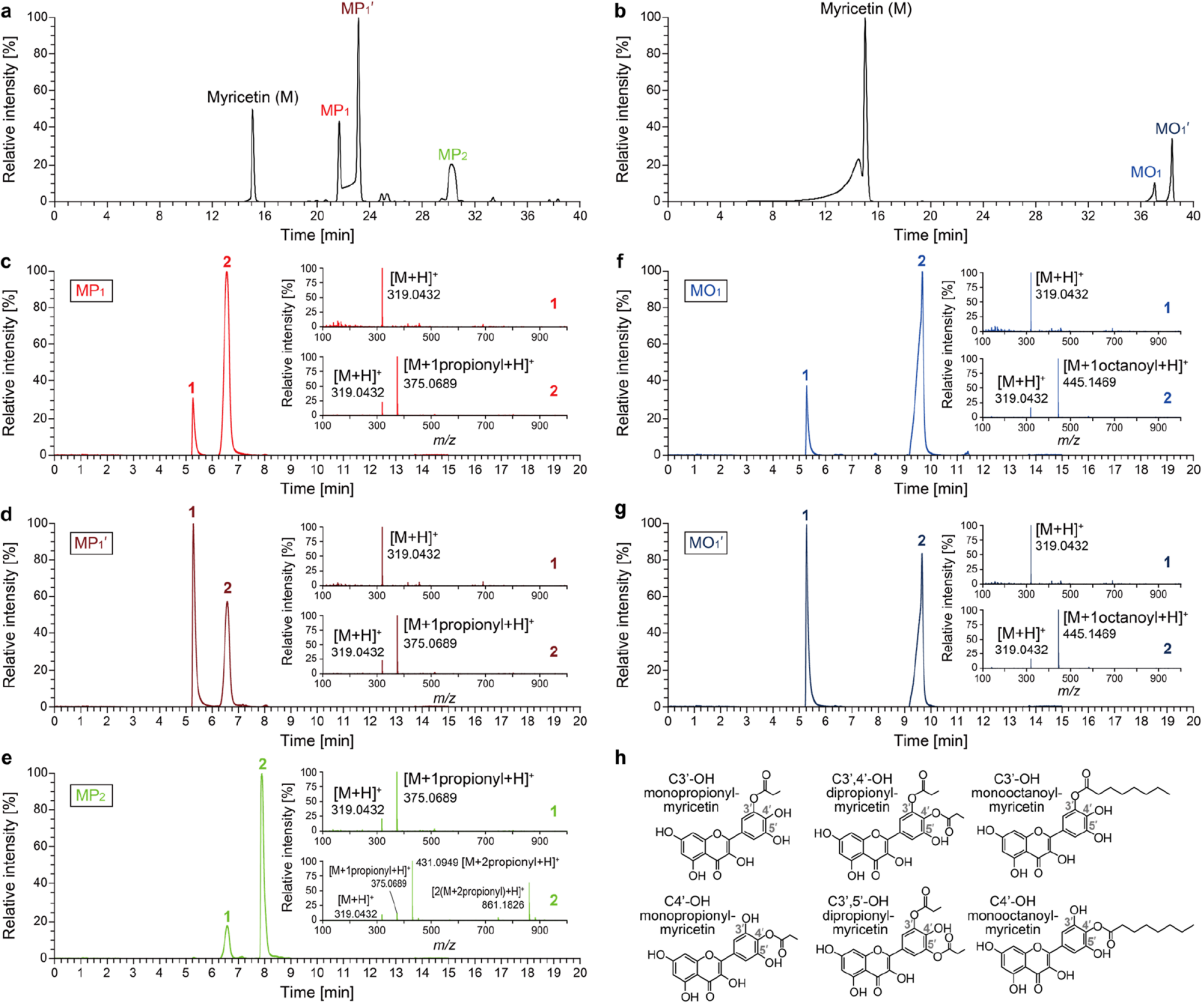
Purification and identification of acylated myricetin products. (**a**, **b**) Representative preparative HPLC chromatograms of acylated myricetin mixtures for separation. Myricetins were converted to (**a**) propionylated myricetin mixture including MP_1_, MP_1_′ and MP_2_ and (**b**) octanoylated myricetin mixture including MO_1_ and MO_1_′ in the presence of TMA. (**c**–**g**) UPLC chromatograms and MS/MS spectra for identification of separated acyl myricetins: (**c**) MP_1_, (**d**) MP_1_′, (**e**) MP_2_, (**f**) MO_1_, and (**g**) MO_1_′. UV/Visible chromatograms were obtained at wavelength 374 nm and positively electrospray-ionized mass (*m/z*) spectra for each fraction are shown (inset). (**h**) Determined structures of C3′-OH monopropionyl-myricetin, C4′-OH monopropionyl-myricetin, C3′,4′-OH dipropionyl-myricetin, C3′,5′-OH dipropionyl-myricetin, C3′-OH monooctanoyl-myricetin, and C4′-OH monooctanoyl-myricetin.

To identify acylated myricetins, we synthesized product mixtures, followed by separation using a preparative high-performance liquid chromatography (HPLC) for purification. The propionylation chromatogram represented four distinct peaks observed at retention times (*t*_r_) of 15.0, 21.6, 23.1, and 30.2 min, which were designated as M, MP_1_, MP_1_′ and MP_2_, respectively (Fig. 3a). This peak profile was also found in the propionylation reaction catalyzed by CaLB (Fig. S2). As for octanoylation, three distinct peaks were observed at *t*_r_ of 15.0, 37.0, and 38.3 min, which were designated as M, MO_1_, and MO_1_′, respectively (Fig. 3b). These fractions were collected, dried, and subjected to further analysis using ultra-high performance liquid chromatography (UPLC)-MS/MS and NMR spectroscopy.

Each fraction was first identified using the UPLC-MS/MS. MP_1_ fraction showed two peaks at *t*_r_ of 5.3 and 6.6 min in the chromatogram, which had 319.0432 and 319.0432/375.0689 m*/z*, respectively, in the MS/MS analysis (Fig. 3c). These values correspond to the masses of the positively charged myricetin ([M + H]^+^, 318.2351 g mol^−1^) and monopropionyl-myricetin ([M + 1propionyl + H]^+^, 374.2976 g mol^−1^), respectively. Although UPLC chromatogram and MS/MS spectra showed signals of free myricetin in the MP_1_ fraction (Fig. 3c and Fig. S3a), any free myricetin peaks such as a chemical shift at 12.50 ppm corresponding to C5′-OH were not observed in the ^1^H NMR spectrum (Fig. S4b and Table S1), strongly implying that deacylation occurred due to high temperature in the column during mass analysis. Similarly, MP_1_′ fraction also showed two peaks at *t*_r_ of 5.3 and 6.6 min, which had *m/z* values of 319.0432 and 319.0432/375.0689, respectively (Fig. 3d and Fig. S3a). Again, ^1^H NMR spectrum of the MP_1_′ fraction showed the sole aspect for monopropionyl-myricetin, not free myricetin (Fig. S5a and Table S1). Through these data, we concluded that the MP_1_ and MP_1_′ fraction consisted only of monopropionyl-myricetin.

In the UV/Vis chromatogram of the MP_2_ fraction (Fig. 3e), two peaks were observed at the *t*_r_ of 6.6 and 7.9 min, which had *m/z* values of 319.0432/375.0689 and 319.0432/375.0689/431.0949/861.1826, respectively. The *m/z* values 431.0949 and 861.1826, stand for the masses of the dipropionyl-myricetin (431.3679 g mol^−1^) and its biflavonoid (a compound combined with two dipropionyl-myricetins, 861.7279 g mol^−1^), respectively. Despite free myricetin MS peak in the chromatogram, ^1^H NMR spectrum (Fig. S4c and Table S1) indicated that the MP_2_ fraction contained only dipropionyl-myricetin as the cases of MP_1_ and MP_1_′. It has been shown that various ions can be detected during electrospray-ionized mass spectrometry even when a pure chemical is under analysis^25^.

The two peaks of MO_1_ fraction at *t*_r_ of 5.3 and 9.7 min had *m/z* values of 319.0432 and 319.0432/445.1469, respectively (Fig. 3f). The *m/z* value of 445.1469 corresponded to the mass of the monooctanoyl-myricetin ([M + 1octanoyl + H]^+^, 444.4295 g mol^−1^). As in the cases of propionyl-myricetins, free myricetin seen in MS spectrum was not observed in the ^1^H NMR spectrum, indicating that the MO_1_ fraction was exclusively composed of monooctanoyl-myricetin (Fig. S4d and Table S1). The MO_1_′ fraction showed almost the same UPLC chromatogram, *m/z* values (Fig. 3g) and ^1^H NMR spectrum as those for the MO_1_ fraction (Fig. S5b and Table S1), suggesting that MO_1_′ has the same molecular weights as MO_1_.

Next, chemical structures of the compounds present in the MP_1_, MP_1_′, MP_2_, MO_1_, and MO_1_′ fractions were determined with ^1^H/^13^C NMR spectra (Figs. S4–S6). Chemical shifts in the spectra were assigned (Tables S1 and S2) based on HMBC and HSQC spectra (Figs. S7 and S8, respectively). Monopropionyl-myricetin of the MP_1_ fraction was not a single compound but comprised of two regioisomer acyl forms: one acylated on hydroxyl group at C3′ in the B-ring of the myricetin (i.e., C3′-OH), and the other acylated on C4′-OH (according to Figs. S4b and S6b). The occupied portion of each monopropionylated form was determined to be 37.5% and 62.5% for acylation at C3′-OH and C4′-OH, respectively, based on the integral values for C1″ in ^13^C NMR spectrum (Fig. S6b). In the same manner, MP_1_′ fraction turned out to contain 41.2% of C3′-OH acylation and 58.8% of C4′-OH form (Fig. S5c). Both MS/MS analyses and NMR results suggested that the MP_1_ and MP_1_′ fractions are practically the same in terms of chemical structure and composition. Different *t*_r_ in the preparative chromatogram between the MP_1_ and MP_1_′ fractions (21.6 and 23.1 min, respectively) is likely based on simple peak splitting phenomenon. Reasons for this phenomenon might be partial column contamination, plugged frit, column void, or injection solvent effect (injecting in a solvent more nonpolar than the starting mobile phase). The same splitting was observed when the MP_1_ or MP_1_′ isolate diluted in acetone was reanalyzed, suggesting the injection solvent effect is a probable reason for the peak splitting.

NMR analyses revealed that MP_2_ fraction contained 47.8% of dipropionylated myricetin with acylation on C3′,4′-OH and 52.2% for C3′,5′-OH (Fig. S6c). For monooctanolyated products, it was shown that MO_1_ fraction presented 41.7% of acylation on C3′-OH and 58.3% of acylation C4′-OH (Fig. S6d), and acylation percentages on C3′-OH and C4′-OH for MO_1_′ fraction were 39.2% and 60.8%, respectively (Fig. S5d). Thus, these data suggest that MO_1_ and MO_1_′ fractions contain virtually the same chemical composition although two peaks were detected in the UPLC due to peak splitting. Meanwhile, we tried to analyze the structure of products after the enzymatic reaction. However, unfortunately we did not obtain enough amount of samples for structural analyses including NMR because the yield for production was too low and the purification was too difficult due to presence of impurities. Indeed, even though we barely prepared enough amount of samples for NMR, we were not able to acquire clear bands in the spectra.

In summary, MS/MS and NMR results suggested that MP_1_ and MP_1_′ had the same chemical composition with ~ 40% of C3′-OH monopropionyl-myricetin and ~ 60% of C4′-OH monopropionyl-myricetin (Fig. 3h and Table S3). MP_2_ fraction was the mixture in which C3′,4′-OH and C3′,5′-OH dipropionyl-myricetins were present in approximately equal proportions. Additionally, the MO_1_ and MO_1_′ fractions showed ~ 40% and ~ 60% of products monooctanoylated at C3′-OH and C4′-OH, respectively. For both MP_1_/MP_1_′ and MO_1_/MO_1_′, it is thought that the larger amounts of products monoacylated at C4′-OH than at C3′-OH resulted from lowest Gibbs free energy of deprotonation (*ΔG*_de_; 0.0 kcal mol^−1^) for the hydroxyl group at C4′ among the six hydroxyl groups in the myricetin^26^. To test the subsequent physicochemical property, functional activity, and toxicity, we used the mixtures of MP_1_ and MP_1_′ fractions for the monopropionyated myricetin sample, and mixtures of MO_1_ and MO_1_′ fractions for the monooctanoyl-myricetin sample, based on the fact that they are identical compounds, only slightly different in composition. The overall composition of the MP_1_ mixture for further testing was 39.3% and 60.7% monopropionylated at C3′-OH and C4′-OH, respectively (Table S3). The composition of the MP_2_ mixture was the same as that of the MP_2_ fraction. The MO_1_ mixture was composed of 40.5% and 59.5% monooctanoylated at C3′-OH and C4′-OH, respectively.

### Physicochemical characterization of the acyl myricetins

Acylation improves the lipophilicity and physicochemical stability of flavonoids^11^. Based on their log*P* values, the lipophilicity for myricetin, MP_1_, MP_2_, and MO_1_ mixtures was determined as 1.34 ± 0.10, 2.21 ± 0.16, 2.42 ± 0.23, and 2.76 ± 0.29, respectively. This result suggests that MP_1_, MP_2_, and MO_1_ mixtures might be 7.4-, 12.0-, and 26.3-times more lipophilic than free myricetin itself, respectively. Specifically, MO_1_ represented the highest superiority in lipophilicity as compared to the other tested compounds, showing 3.5- and 2.2-fold more lipophilic properties than MP_1_ and MP_2_, respectively. It is thought that this is likely attributed to longer hydrocarbon chain length in octanoyl group compared to propionyl group.

**Figure 4:**
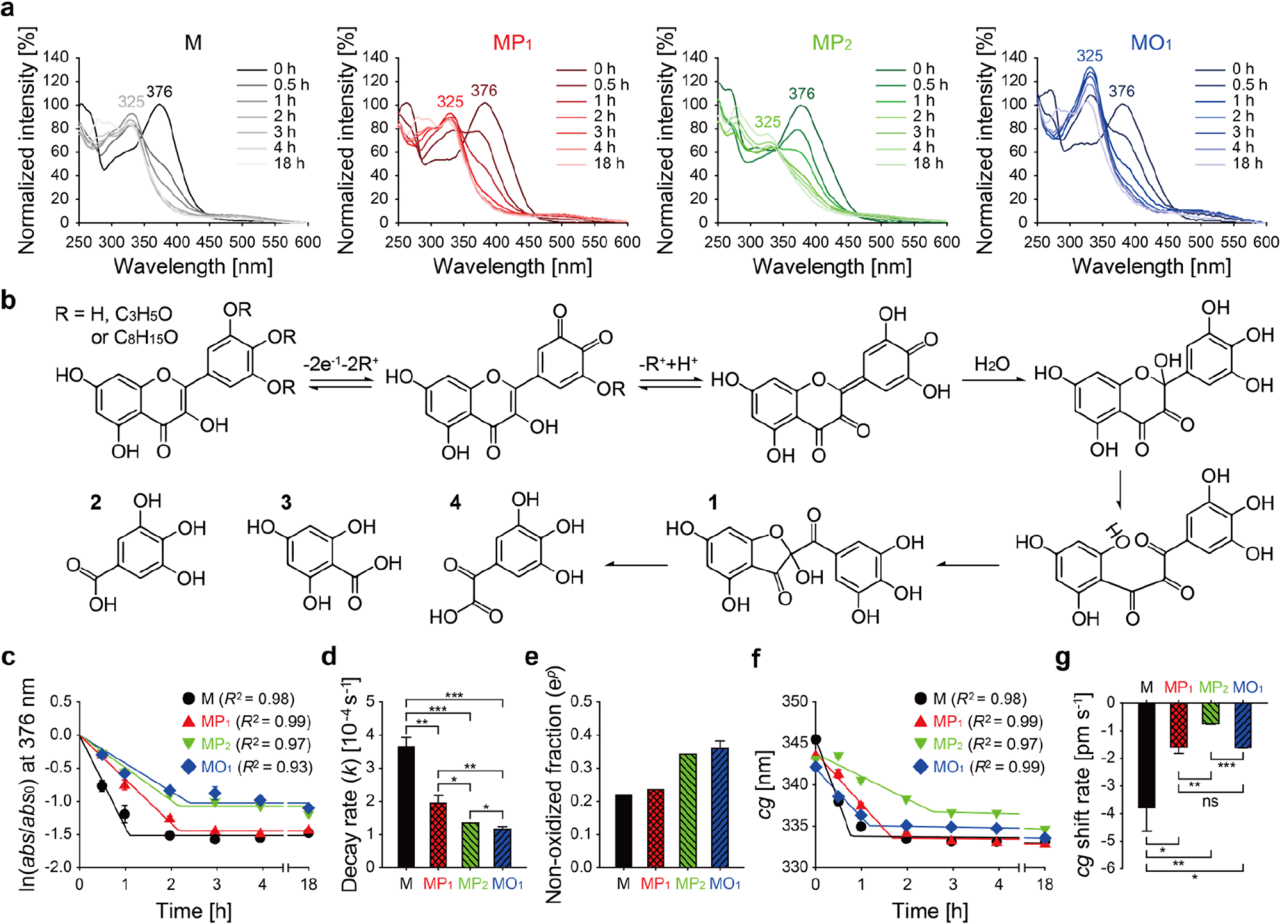
Physicochemical stability of acyl myricetins. (**a**) Changes in the absorbance spectra of M, MP_1_, MP_2_ and MO_1_ after incubation in aqueous condition. Acyl myricetins, dissolved in DMSO, were diluted into PBS and immediately incubated for 18 h (pH 7 and 37 °C), followed by measurement of UV/Vis-light absorbance spectra at a wavelength ranged from 250 to 600 nm. (**b**) Expected schematic representation of the oxidation procedure for myricetin and its acylated forms (compounds 1‒4, expected oxidation products). (**c**) Exponential decay-plateau fitting curves [$\mathrm{ln}(abs/{abs}_{0})=-kt \left(0\le t<{t}_{\mathrm{p}}\right)=p ({t}_{\mathrm{p}}\le t)$; $abs$, absorbance (wavelength, 376 nm) at the incubation time ($t$); ${abs}_{0}$, absorbance at initiation of the incubation; $k$, decay rate constant; $p$, constant on the plateau] of the tested compounds for the absorbance spectra. (**d**) Decay rate constants ($k$) and (**e**) non-oxidized fraction ratios (${\mathrm{e}}^{p}$) determined from the decay fitting curves. (**f**) Shifting of the center of gravity for the absorbance spectra, which was determined using the two segment linear fitting curves [$cg={k}_{1}t+{cg}_{1}\left(0\le t<{t}_{1}\right)={k}_{2}t+{cg}_{2} ({t}_{1}\le t)$]. (**g**) The $cg$ shift rates determined from the two segment linear fitting curves. Student’s *t*-test: ns, non-significant, *p* > 0.05; *, *p* < 0.05; **, *p* < 0.01; and ***, *p* < 0.001 (**d**, **g**). Data are expressed as the mean ± s.d (**c**–**g**).

The physicochemical stability of myricetin and its acylation derivatives against oxidation in aqueous solution was investigated through the UV/Vis-light absorbance spectra (Fig. 4a). At the initial incubation point, the absorbance bands in the spectra of all compound mixtures were observed at 376 nm in an aqueous environment (pH 7 and 37 °C), representing the signal for the double bond between the C2 and C3 atoms^26^. In a manner similar to that observed for quercetin in a neutral pH environment, myricetin is di-deprotonated, not only at C4′-OH, the most acidic hydroxyl group, but also at C3′-OH^26^. Thus, the acyl myricetin mixtures can be deprotonated or deacylated at C4′-OR, C3′-OR, and C5′-OR, as shown in Fig. 4b. Oxidation of myricetin and its acyl mixtures can occur, at this point, with the formation of quinone intermediates^27^. The quinone intermediates are transformed by the addition of H_2_O, and subsequently the double bond between C2 and C3 atoms disappears, where the C-ring of the transformed intermediates can be opened and then reclosed as compound 1 [2-(2′,3′,4′-trihydroxybenzoyl)-2,4,6-trihydroxybenzofuran-3(2*H*)-one]^28^. As compound 1 was formed, reduced absorbance at 376 nm and increased absorbance at 325 nm were evident in all of the spectra (Fig. 4a). Compound 1 can be further degraded into compounds 2 (2,3,4-trihydroxybenzoic acid), 3 (2,4,6-trihydroxybenzoic acid), and 4 [2-(2,3,4-trihydroxyphenyl)-2-oxoacetic acid]^29^, resulting in decreased absorbance at 325 nm. Hence, rate ($k$) and extent ($1-{\mathrm{e}}^{p}$) of the decay/oxidation were estimated based on the absorbance at 376 nm, representative of the C2=C3 double bonds of myricetin, MP_1_, MP_2_, and MO_1_ (Fig. 4c).

The $k$ value of myricetin, 3.7 × 10^−4^ s^−1^, was the highest among the samples, suggesting that myricetin is the most vulnerable to rapid oxidation (Fig. 4d). The $k$ values of MP_1_ and MP_2_ mixtures were 2.0 × 10^−4^·s^−1^ and 1.3 × 10^−4^·s^−1^, which were ~ 1.9- and 2.7-fold lower than that of myricetin, respectively. With this result, we realized that a higher level of acylation potentially elicits the ability to reduce the rate of oxidation as described^30^. Notably, the $k$ value of the MO_1_ mixture was 1.2 × 10^−4^·s^−1^, ~ 3.1-fold lower than that of myricetin, indicating the excellent protection against oxidation among the acylated compounds, despite only having a single acylation. Similarly, non-oxidized fraction result also showed the same tendency based on ${\mathrm{e}}^{p}$ values (Fig. 4e), providing a probability that the chain length of the vinyl ester used for acylation might be a critical factor in minimizing the oxidation of flavonoids such as myricetin. This result also demonstrates that MO_1_ mixture exhibited the highest resistance to oxidation, which is consistent with decay rate results. Based on the fact that the detection of the absorbance band shift allows the measurement of the oxidation for compounds, we determined the $cg$ shift rate by using $cg$ values (Fig. 4f). As shown in Fig. 4g, myricetin showed the most rapid $cg$ shift of − 3.8 pm·s^−1^, which was ~ 2.4-, 5.1-, and ~ 2.3-times faster than those for MP_1_, MP_2_, and MO_1_ mixtures, respectively. This also suggests the slowest $cg$ shift—for the MP_2_ mixture—could be attributed to structural properties of the MP_2_ mixture acylated to higher levels than that of MP_1_ or MO_1_. From the data studying physicochemical characterization of acyl myricetins, it is most likely that lipophilicity and chemical stability can be significantly enhanced, depending on the length and number of carbon chains for the adopted vinyl esters. The enhanced chemical stability by acylation is meaningful for myricetin-using food, cosmetic, and pharmaceutical products, as the color change reduction and shelf life increasing during the storage and use. In addition, the increased lipophilicity can enlarge the loading amount of myricetin into lipid-based formulations conventionally used for foods, cosmetics, and pharmaceuticals, which might improve bio-efficacies of the products.

### Inhibition of neurotransmitter release and biocompatibility of the acyl myricetins

**Figure 5:**
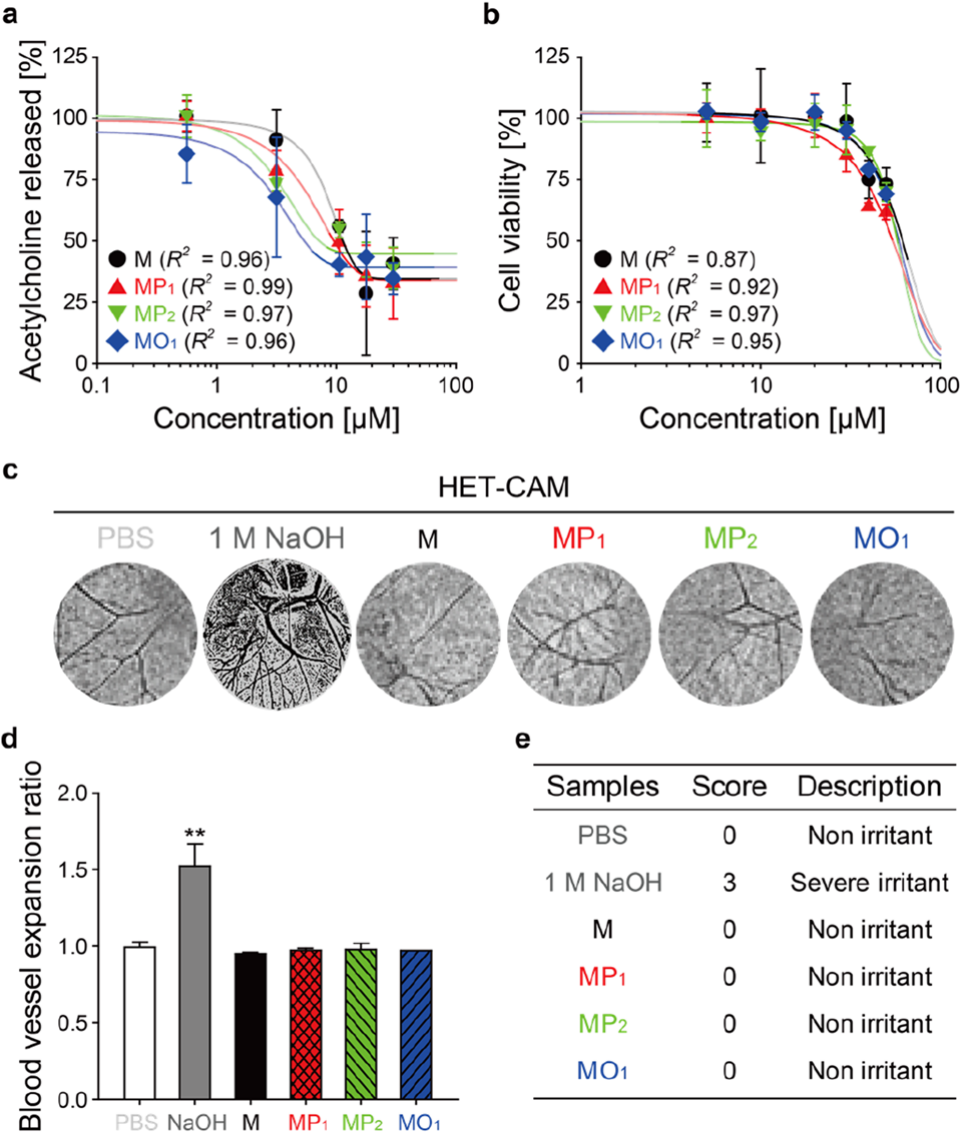
Comparison of inhibitory activity of acyl myricetins on neurotransmitter release and their safety evaluation. (**a**) Inhibition of acetylcholine release from differentiated PC-12 cells by M, MP_1_, MP_2_ and MO_1_. (**b**) Cytotoxicity of acyl myricetins on PC-12 cells, determined by neutral red uptake assay. (**c**) Images of the HET-CAM test after treatment with 2 mL of PBS, 1 M NaOH aqueous solution, and 1 mg·mL^−1^ solutions of M, MP_1_, MP_2_ and MO_1_ mixtures, which were evaluated using ImageJ to determine the area of blood vessels. (**d**) Ratios of the black pixel area fraction (%Area) in the ImageJ images between after and before the sample solution treatments (blood vessel expansion ratio). Student’s *t*-test: **, *p* < 0.01. (**e**) Scores obtained from the HET-CAM test results. Data are expressed as the mean ± s.d (**a**, **b**, **d**).

Myricetin is able to inhibit the formation of the neuronal SNARE complex which mediates the release of neurotransmitters at synaptic clefts^20^. Acetylcholine released from differentiated PC-12 cells was quantified to assess the inhibitory effects of the acyl myricetins on neuroexocytosis (Fig. 5a). The median effective concentrations (EC_50_) were 5.36, 3.84, and 3.58, and 2.51 μM for free myricetin, MP_1_, MP_2_ and MO_1_, respectively (Table 1). There was no significant difference between free myricetin and samples (MP_1_ and MP_2_) other than MO_1_ in EC_50_. Interestingly, this result corresponds with those of the log*P* values and oxidation studies (Fig. 4d,e), suggesting that the superior inhibitory effect of MO_1_ is probably attributed to both lipophilicity and chemical stability enhanced by acylation. Indeed, the increased lipophilicity elevates passive diffusion/permeability of various flavonoids through membranes^31,32^, and the improved chemical stability can increase the non-oxidized fraction.

**Table 1:** EC_50_, CC_50_ and EC_50_/CC_50_ values of M, MP_1_, MP_2_, and MO_1_ mixtures in differentiated PC-12 cells.

Samples	EC_50_ (μM)	CC_50_ (μM)	EC_50_/CC_50_
M	5.36 ± 0.20 b	66.4 ± 10.9 a	0.08 ± 0.01 b
MP_1_	3.84 ± 1.08 ab	54.6 ± 0.6 a	0.07 ± 0.02 ab
MP_2_	3.58 ± 0.75 ab	58.4 ± 1.6 a	0.06 ± 0.01 ab
MO_1_	2.51 ± 0.89 a	59.0 ± 1.4 a	0.04 ± 0.01 a

Different letters in each column are significantly different (Tukey’s test, *p* < 0.05).

Next, the cytotoxicity of acyl myricetins was evaluated via neutral red uptake (NRU) assay using PC-12 cells (Fig. 5b). The median cytotoxic concentrations (CC_50_) were determined as 54.6, 58.4, and 59.0 μM for MP_1_, MP_2_, and MO_1_, respectively, which were not significantly different from that of myricetin (65.4 μM) (Table 1). With the EC_50_ values obtained in Fig. 5a, we calculated the EC_50_/CC_50_, which is an indicator of the therapeutic window, and found that MO_1_ mixture showed the lowest value (0.04) among the tested chemical compounds, presenting a widest therapeutic window of MO_1_ than those for M, MP_1_, and MP_2_ (0.08, 0.07, and 0.06, respectively). In order to further evaluate safety and biocompatibility for acyl myricetins, HET-CAM test was performed, which is an excellent in vivo alternative approach to animal testing. The results demonstrated that all acyl myricetins did not exhibit any irritancy effects such as hemorrhage, lysis of blood vessels, or intravasal coagulation as compared with controls (Fig. 5c‒e and Fig. S9). Through these results, we concluded that the acylation possibly confers the improved neuroexocytosis-inhibitory effect to the myricetin while adding no toxicity, suggesting that acylated myricetins, particularly MO_1_, might be promising compounds for industrial applications such as facial cosmetic and pharmaceutical fields.

**Figure 6:**
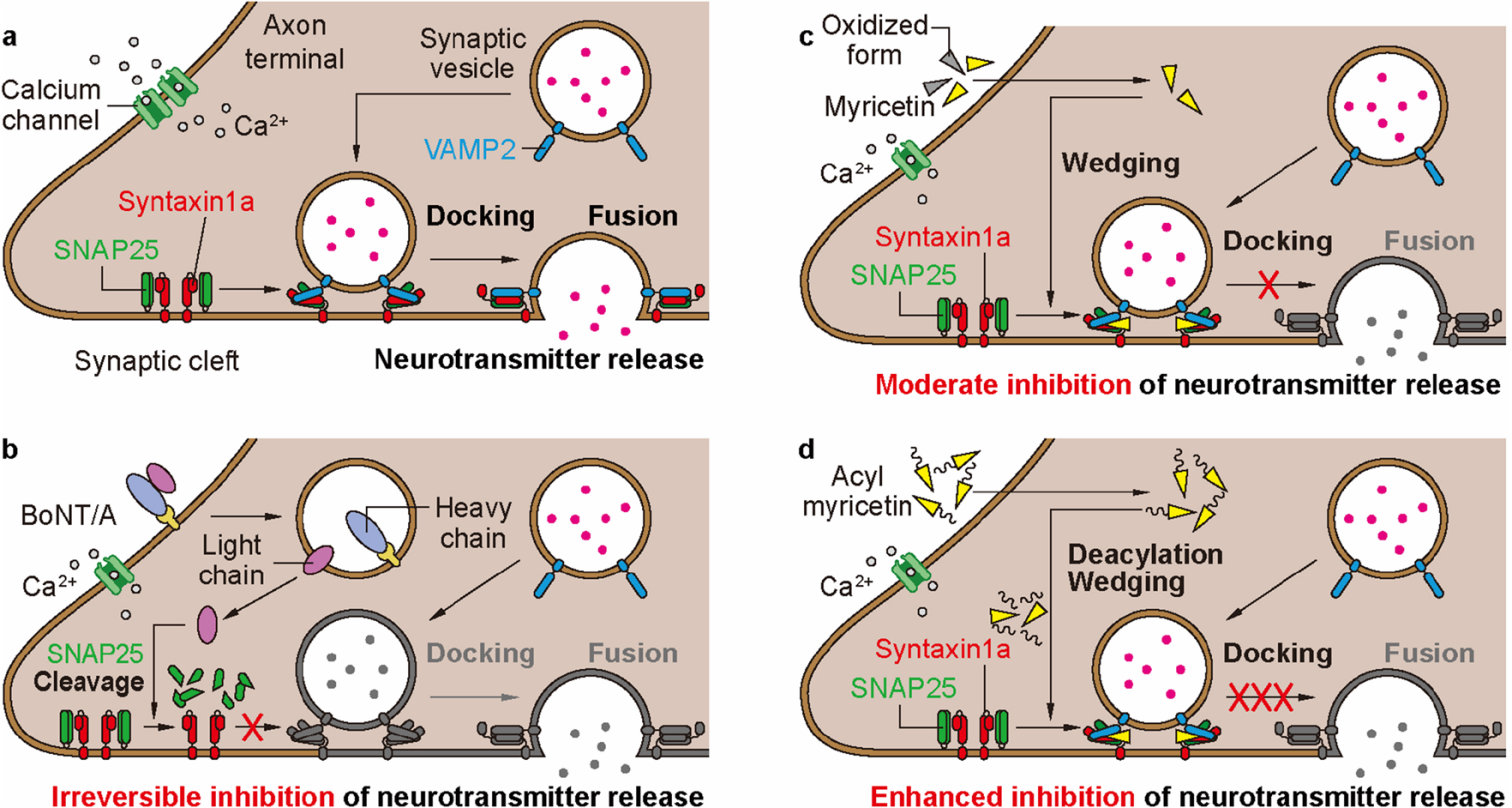
Proposed mechanism-of-action of acyl myricetins showing BoNT/A-like activity for inhibition of neurotransmission. (**a**) Neurotransmitter release by SNARE-mediated membrane fusion. At the axon terminal, synaptic vesicle is recruited to active zone for ‘docking’, where t-SNARE proteins on the presynaptic membrane, Syntaxin 1a and SNAP25, and v-SNARE protein VAMP2 on the synaptic vesicle form a SNARE complex. With calcium influx, membrane fusion occurs, allowing neurotransmitter to release into the synaptic cleft. (**b**–**d**) Inhibitory activity of acyl myricetins on neurotransmission compared to BoNT/A and parental myricetin. Unlike BoNT/A, which inhibits neurotransmission by cleaving SNAP25 after internalization into the cell (**b**), (**c**) myricetin and (**d**) its acylated derivatives intercalates and wedges into the inner layer of the SNARE complex during its zippering process, eventually leading to inhibition of neurotransmitter release. It is noteworthy that the enhanced inhibitory effect of acylated myricetin is likely due to the improved oxidation resistance and lipophilicity-based penetration property compared to myricetin (**d**). *SNAP25* synaptosomal-associated protein 25 kDa, *VAMP2* vesicle-associated membrane protein 2, *BoNT/A* botulinum neurotoxin type-A.

In normal conditions, neurotransmitters such as acetylcholine are released from synaptic vesicles to synaptic cleft via the SNARE complex formation (Fig. 6a). A light chain of endocytosed BoNT/A cleaves SNAP25, prevent the SNARE complex formation, and irreversibly inhibit the neurotransmitter release (Fig. 6b). Whereas some myricetin molecules absorbed are degraded in neutral cytoplasm conditions, the rest of the molecules can hinder the SNARE complex formation and moderately inhibit the neurotransmitter release (Fig. 6c). In this study, we show that acyl myricetins are physicochemically more stable at neutral pH and 37 °C than free myricetin, which increases the amount of the non-oxidized fraction. Nevertheless, the lipophilicity is the most important factor rather than stability because MP_2_ exhibiting comparable stability with MO_1_ has lower inhibitory activity toward acetylcholine release than MO_1_. Due to their higher lipophilicity, the non-oxidized fractions of these esters are expected to more effectively diffuse into the cells through the neuronal membrane, where the acyl myricetins are likely susceptible to the deacylation (Fig. 6d) as reported for the other flavonoid^33^. Then, the intracellular myricetin, unlike BoNT, would inhibit the formation of the SNARE complex and subsequent membrane fusion by intercalating in the middle of SNARE zippering, eventually blocking the neurotransmitter release into the synaptic cleft.

Myricetin has been known as a flavonol having multifuntionality of anti-oxidant^14^, anti-cancer^15,16^, neuroprotective^17^, and membrane fusion-arresting^19,20^. In this study, acyl myricetins, particularly MO_1_, showed an enhanced inhibition effect on a neurotransmitter release owing to the higher lipophilicity than myricetin. If the enhanced inhibition effect on a neurotransmission is due to an increased simple diffusion through cell membrane by the higher lipophilicity, acyl myricetins might also show an improvement of other diverse health beneficial effects reported previously^18^, at least in cell level. Additionally, the increased lipophilicity can enlarge the loading amount of myricetin into conventional lipid-based formulations for foods, cosmetics, and pharmaceuticals. Consequently, acyl myricetins as a multifunctional flavonol acylated can be applied to foods, cosmetics, and pharmaceuticals more widely and efficiently than myricetin.

## Conclusions

Among acyl myricetins synthesized in this study, the MO_1_ was the most lipophilic and physicochemically stable in aqueous conditions. Additionally, the MO_1_ efficiently inhibited the acetylcholine release with the smallest EC_50_ despite the low cytotoxicity to neuronal PC-12 cells. For the HET-CAM results, all the acyl myricetins exhibit no ocular toxicity, which demonstrated the biocompatibility as safe as it can be applied to some commercial fields, including facial cosmetic industry. Moreover, acyl myricetins may engage multifuctionality, better than myricetin as a multifunctional flavonol in medicine including anti-cancer, neuroprotective, and membrane fusion-arresting bioactivities. In conclusion, acyl myricetins and their synthesis method may hold a great promise for development of stable, effective, and safe feedstocks for foods, pharmaceuticals, and cosmetics, to lessen the symptoms associated with excessive neurotransmission.

## Methods

### Materials

Myricetin and TMA were purchased from Sigma Aldrich Co. (St. Louis, MO, USA). VP and VO were obtained from Tokyo Chemical Industry (Tokyo, Japan). CaLB (Novozym 435) was procured from Novozymes (Bagsvaerd, Denmark). Roswell Park Memorial Institute (RPMI) 1640 media, antibiotic–antimycotic, horse serum, and fetal bovine serum (FBS) were obtained from GIBCO/Invitrogen (Grand Island, NY, USA). All chemicals were of analytical reagent grade.

### Acylation of myricetin

All acylation reactions were performed in micro-tubes containing acetone, shaken at 700 rpm at 50 °C in a heating drybath (S08040, Thermo Scientific, OH, USA). For the acylation without catalyst, the myricetin concentration was 0.3 mg·mL^−1^ and the molar ratio of myricetin:vinyl ester (either VP or VO) was 1:500. For CaLB-catalyzed reactions, the composition of all reactants was the same as that used for the non-catalyzed reaction, except for the presence of 20 mg·mL^−1^ of CaLB. In the reactions catalyzed by TMA (10 mol% myricetin), the concentration of myricetin was 5 or 20 mg·mL^−1^ and the molar ratio of myricetin-to-vinyl ester was either 1:10 or 1:50. At predetermined times, the samples were withdrawn from the reaction vessel and appropriately diluted with acetone for analysis.

### Analysis of the acyl myricetins

The acylated myricetin mixtures were analyzed using an HPLC system (Waters 1525; Waters Corporation, Milford, MA, USA), equipped with a Synergi Hydro-RP 80 Å LC column (4 μm, 4.6 × 250 mm; Phenomenex, Torrance, CA, USA) and UV/Vis light detector (Waters 2487; wavelength 360 nm; Waters Corporation). Column temperature was maintained at 37 °C, the flow rate of the mobile phase was 1.3 mL·min^−1^, and the injection volume of samples was 20 μL. The mobile phase was a mixture of 0.05% (v/v) acetic acid in double-deionized water (DDW; A) and 0.1% (v/v) acetic acid in acetonitrile (B) for a total running time of 23 min, and the following proportions of solvent B were used for elution: 0 → 3 min, 30 → 50% (v/v); 3 → 5 min, 50 → 90% (v/v); 5 → 18 min, 90% (v/v); and 18 → 23 min, 90 → 30% (v/v). The amounts of acylated products shown in Figs. 1 and 2 were quantified based on the areas of peaks that appeared in the specific range of *t*_r_ (myricetin, ~ 3.0–4.0 min; MP_1_, ~ 5.0–5.5 min; MP_2_, ~ 6.0–6.5 min; MP_>2_, ~ 6.8–7.2 min; MO_1_, ~ 6.5–7.1 min; MO_2_, ~ 9.0–10.2 min) in typical HPLC chromatograms (Fig. S10).

### Separation of the acyl myricetins

The acylated myricetin mixtures were first concentrated by acetone-evaporation in a rotavapor (N-1200A; Eyela Co., Tokyo, Japan) and re-dissolved with acetone, at an appropriate concentration. To separate acyl myricetins, the mixtures were loaded on a Kromasil 100-5 C18 Column (5 μm, 21.2 × 250 mm; Eka Nobel AB, Bohus, Sweden), equipped with a preparative HPLC system (LC-8A; Shimadzu Co., Kyoto, Japan) and analyzed using a UV/Vis light detector (Waters 2996; Waters Corporation). The linear gradient of the mobile phase (27 °C; flow rate of 8 mL·min^−1^), mixed with acetonitrile and 0.05% (v/v) trifluoroacetic acid in DDW, was increased from 30 to 70% (v/v) based on acetonitrile. The collected eluates were then powdered using the rotavapor (N-1200A; Eyela Co.). Typical chromatograms are shown in Fig. S2.

### UPLC-UV/Vis light detector-mass/mass

The UPLC analyses were conducted using an Accelar UPLC module system (Waters Corporation) connected to a UV/Vis light detector (Waters 2996; wavelength 374 nm; Waters Corporation) and an LTQ orbitrap XL (Thermo Fisher Scientific Inc., MA, USA). Briefly, the separated acyl myricetin solutions (2 μL) were loaded on an Acquity UPLC BEH C18 column (1.7 μm, 2.1 × 100 mm; Waters Corporation) at 40 °C with a flow rate of 0.4 mL·min^−1^. The mobile phase included a mixture of 0.1% (v/v) formic acid in DDW (A) and 0.1% (v/v) formic acid in acetonitrile (B) for a total running time of 20 min. The following proportions of solvent B were used for elution: 0 → 3 min, 5% (v/v); 3 → 10 min, 5 → 30% (v/v); 10 → 11 min, 30% (v/v); 11 → 14 min, 30 → 100% (v/v); 14 → 15 min, 100% (v/v); 15 → 16 min, 100 → 5% (v/v); and 16 → 20 min, 5% (v/v). The effluent from the UPLC was positively electrospray-ionized into the LTQ orbitrap XL (capillary temperature, 350 °C; capillary voltage, 20 V; spray voltage, 3.5 kV). Survey full-scan mass spectra (*m/z*, 150–1500) were acquired using the orbitrap analyzer.

### NMR spectroscopy

Proton/carbon nuclear magnetic resonance (^1^H/^13^C NMR) spectra of the separated myricetin esters, dissolved in dimethyl sulfoxide (DMSO; 25 mg·mL^−1^), were obtained using an Avance III 700 MHz spectrometer (Bruker Biospin, Rheinstetten, Germany) at 700.65/176.13 Hz (for ^1^H/^13^C NMR) at an ambient temperature of 25‒29 °C. Chemical shifts were assigned, based on heteronuclear multiple bond correlation (HMBC) and heteronuclear single quantum coherence (HSQC) spectra.

### Determination of partition coefficients

Values for log*P* were determined to quantify the lipophilicity or hydrophilicity of the separated acyl myricetins, using the previously reported method with slight modifications^12^. After the addition of 1-octanol (500 μL) into 2 mL micro-tubes containing 1 mg of the separated acyl myricetins, the micro-tubes were vortexed for 1 min, followed by sonication (bath-type) for 5 min, and then 500 μL of DDW was added to the tubes. Next, the mixed solutions in the tubes were vortexed again for 5 min and phase-separated by centrifugation (3000 relative centrifugal force for 10 min at 25 °C). The 40 μL of upper layer (1-octanol) and 100 μL of lower layer (DDW) were collected and diluted with 1.96 mL of 1-octanol and 100 μL of DMSO, respectively. The solutes dissolved in the upper and lower layers were quantified based on the standard curves obtained from the UV/Vis-light absorbance spectra (250‒600 nm) using a microplate reader (Synergy H1; BioTek Instruments Inc., VT, USA; measuring interval of 3 nm, at 25 °C). Finally, the log*P* values were calculated as $log ({c}_{octanol}/{c}_{DDW}),$ where ${c}_{octanol}$ and ${c}_{DDW}$ are the concentrations of the solutes in the upper and lower layers, respectively.

### Stability assessment

To assess their stability in an aqueous environment at pH 7 and 37 °C, UV/Vis-light absorbance spectra of the separated acyl myricetins were obtained using the Synergy H1 (BioTek Instruments Inc.; measuring interval of 3 nm, 25 °C), at a wavelength-range of 250‒600 nm^34^. One hundred microliters of the separated esters (20 μg·mL^−1^), dissolved in DMSO, were mixed in a micro-tube (2 mL) with 1.9 mL of phosphate buffered saline (PBS), pre-adjusted to pH 7 with 1 M HCl aqueous solution. After covering with a lid, the mixture was immediately incubated for 18 h in a heating shaking drybath (S08040; Thermo Scientific) at 37 °C, shaken at 700 rpm. At predetermined times (0, 0.5, 1, 2, 3, 4, and 18 h), 200 μL of the incubated mixtures were transferred into wells of a 96-well microplate, and their UV/Vis absorbance spectra were recorded.

The amount of non-oxidized acyl myricetin was determined, based on the absorbance ($abs$) at a wavelength of 376 nm at incubation time ($t$). An exponential decay-plateau fitting method was employed to determine decay rate constant values ($k$, s^−1^) for the acyl myricetins, as follows:

$$ln (abs/{abs}_{0} )=-kt \left(0\le t<{t}_{p}\right)=p ({t}_{p}\le t)$$

where ${abs}_{0}$ is the absorbance at the initiation of incubation and ${t}_{p}$ is the time in which the left side ($-kt$) reaches the plateau constant ($p$). The non-oxidized proportion of the esters, after all the incubation, was described as ${e}^{p}$. Monitoring the shift of specific peaks in the spectra for the acyl myricetins could be important for application in food and cosmetics because this can indirectly indicate color changes in the product, which would be unwelcomed to most of the customers. Therefore, the center of gravity in the spectra of the M esters was determined as follows, in order to monitor the shift of specific peaks

$$Center\,of\,gravity \left(cg\right)=\sum ({abs}_{i}{\lambda }_{i})/\sum ({abs}_{i})$$

where ${abs}_{i}$ is the absorbance at a specific wavelength (${\lambda }_{i}$, 300‒400 nm). Based on the $cg$ estimated during the incubations, the $cg$ shifting rate (${k}_{1}$) was determined using the two segment linear fitting curves as follows:

$$cg={k}_{1}t+{cg}_{1}\left(0\le t<{t}_{1}\right)={k}_{2}t+{cg}_{2} ({t}_{1}\le t)$$

where ${cg}_{1}$ and ${cg}_{2}$ are intercepts of linear curves for the left and right sides, respectively, ${t}_{1}$ is the time that the left side (${k}_{1}t+{cg}_{1}$) takes to become the right side (${k}_{2}t+{cg}_{2}$), and ${k}_{2}$ is an inclination of curve toward the right side.

### Cell culture

PC-12 cells, obtained from Korean Cell Line Bank (KCLB, Seoul, Korea), were cultured in media containing 84% (v/v) RPMI 1640 medium, 10% (v/v) heat-inactivated horse serum, 5% (v/v) heat-inactivated FBS, and 1% (v/v) antibiotic–antimycotic (100×) solution, under humidified conditions (37 °C, 5% CO_2_). The culture medium was refreshed every other day. Prior to further experiments, the viability of the cells in passages 1–5 was assessed using the trypan blue exclusion test.

### Acetylcholine release assay

Harvested PC-12 cells were seeded into 24-well plates coated with poly-d-lysine (2 × 10^5^ cells per well) and grown in the culture media for one day (37 °C, 5% CO_2_). The culture media were then replaced with low serum media (94% (v/v) RPMI 1640 medium, 2.5% (v/v) heat-inactivated horse serum, 2.5% (v/v) heat-inactivated FBS, and 1% (v/v) antibiotic–antimycotic solution) containing 100 ng·mL^−1^ of nerve growth factor (NGF mouse protein 2.5S subunit; GIBCO/Invitrogen) and incubated for 5 days. Then, the media were replaced with the low serum medium containing the acyl myricetins and incubated for 12 h. Next, the acetylcholine released from the cells into the medium was quantified using an Acetylcholine Assay Kit (Fluorometric; Cell Biolabs, Inc., CA, USA) and the Synergy H1 (*λ*_ex_/*λ*_em_, 530 nm/600 nm). Subsequently, using the Kit, the amount of released acetylcholine was determined by subtracting the basal level signal from the signal for sample-treated cells.

### NRU assay

Harvested PC-12 cells (~ 80% confluency per well) were seeded into 96-well plates coated with type I-collagen and grown in the culture media for one day (37 °C, 5% CO_2_). Cells were treated with media containing the acyl myricetins and incubated for 12 h. The media were aspirated, and cells were then incubated for 2 h with 0.33% (w/v) neutral red dissolved in the media. After the incubation, the neutral red media were carefully withdrawn, and cells were rinsed twice with PBS at 37 °C and dried for 1 h. Next, neutral red contained in the dried cells was extracted and solubilized with a solution of 50% (v/v) ethanol, 49% (v/v) DDW, and 1% (v/v) acetic acid, by incubating for 10 min. Absorbance of the neutral red-containing solution at 540 nm was recoded using the Synergy H1. Cell viability was determined based on the absorbance of treated cells compared to that of untreated cells (without acyl myricetins).

### HET-CAM test

The HET-CAM is used for detecting ophthalmic irritancy in vivo^35^. For the HET-CAM, fertilized eggs were obtained from a local market in Korea and incubated for 10 days (37.5 °C and 45% humidity), by maintaining autorotation at 90°·h^−1^ to ensure proper development and viability of the embryos. After incubation, the upper shell was cut in a circular shape, without damaging the inner membrane. Immediately after cutting, the inner membrane was carefully removed with forceps, to avoid injury to the blood vessels, and 2 mL of acyl myricetin solutions were placed on the CAM. Prior to the treatment, the solutions were prepared by mixing 1.9 mL of PBS and 100 μL of DMSO dissolving acyl myricetins, to prevent sedimentation of the crystalline solute. A mixture of PBS (1.9 mL) and DMSO (100 μL), and 1 M NaOH aqueous solution were used as negative and positive controls, respectively. Next, by turning an LED flashlight at the bottom of eggshell, CAM images were obtained 1 min after samples addition.

Based on the CAM images, the severity of any hemorrhage, blood-coagulation, and hyperemia was graded from 0 (no irritation) to 3 (strong irritation) according to a previously developed scoring method^35^. We analyzed the CAM images using ImageJ (http://rsb.info.nih.gov/ij/) to prevent arbitrary judgments with respect to the previous scoring method; then we selected blood vessels in the CAM images, and measured hemorrhage, blood-coagulation, and hyperemia as follows. First, the CAM images were loaded individually into ImageJ, cropped to a fixed size to exclude the shell, and converted to grayscale. The CAM images, grayscaled with red stack, were adjusted using the MidGrey method, and the area fraction (%Area) values of black pixels in the adjusted CAM images were measured and recorded.

### Statistical analyses

All data represented an average of at least three independent experiments or measurements and were reported as averages with standard deviations (s.d.). Kinetic parameters and regression curves were estimated and fitted using a linear/nonlinear regression iteration procedure, using SigmaPlot (V10.0, IBM Co., Armonk, NY, USA). Statistical analyses (Tukey’s test or Student’s *t*-test) were conducted using SPSS Statistics (V23.0, IBM Co., Armonk, NY, USA).

## Supplementary Information

Supplementary Information.

## Data Availability

The datasets used and/or analyzed during the current study available from the corresponding author on reasonable request.
