# Supplementary material for: Synthesis and physicochemical characterization of acyl myricetins as potential anti-neuroexocytotic agents
Source: Sci Rep. 2023 Mar 29;13:5136. doi: 10.1038/s41598-023-32361-6 (PMC10060577; doi:10.1038/s41598-023-32361-6)
Supplement: Supplementary file 1 — Supplementary Information. [file 41598_2023_32361_MOESM1_ESM.pdf]

Synthesis and physicochemical characterization of acyl myricetins as potential anti-neuroexocytotic agents

Sora Cho[1†], Byoungjae Kong[2,3†], Younghun Jung[4], Jonghyeok Shin[5], Myungseo Park[6], Woo-Jae Chung[1,7,8,9]*, Choongjin Ban[10]*, Dae-Hyuk Kweon[1,7,8,9]*

[1]Interdisciplinary Program in BioCosmetics, Sungkyunkwan University, 2066 Seoburo, Suwon, Gyeonggi 16419, Republic of Korea

[2]Center for Nanomedicine, Wilmer Eye Institute, Johns Hopkins University School of Medicine, Baltimore, MD 21231, USA

[3]Department of Ophthalmology, Johns Hopkins University School of Medicine, Baltimore, MD 21231, USA

[4]Wallace H. Coulter Department of Biomedical Engineering, Emory University and Georgia Institute of Technology, 313 Ferst Drive, Atlanta, GA 30332, USA

[5]Synthetic Biology Research Center, Korea Research Institute of Bioscience and Biotechnology (KRIBB), Daejeon 34141, Republic of Korea

[6]Environmental Health Sciences, School of Public Health, University of Minnesota, Saint Paul, MN 55108, USA

[7]Department of Integrative Biotechnology, [8]Institute of Biomolecule Control, and [9]Biologics Research Center, Sungkyunkwan University, 2066 Seoburo, Suwon, Gyeonggi 16419, Republic of Korea

[10]Department of Environmental Horticulture, University of Seoul, 163 Seoulsiripdaero, Dongdaemun-gu, Seoul 02504, Republic of Korea

*Corresponding authors:
Woo-Jae Chung (Email: wjchung@skku.edu), Choongjin Ban (Email: pahncj@uos.ac.kr), and Dae-Hyuk Kweon (Email: dhkweon@skku.edu).

†These authors contributed equally to this study.

Table S1. ^{1}H NMR (DMSO, 700 MHz) chemical shift (*δ*, ppm) of myricetin and acyl myricetins (MP_1, MP_1', MP_2, MO_1, and MO_1').

[1]H location	M	MP_1		MP_1'		MP_2		MO_1		MO_1'	
		C3′ [a]	C4′ [b]	C3′	C4′	C3′,4′ [c]	C3′,5′ [d]	C3′	C4′	C3′	C4′
3OH	9.33 (1H, br s)	9.53	9.61 (1H, s)	9.53	9.61	9.72 (1H, s)	9.85	9.53 (1H, s)	9.61	9.54	9.62
5OH	12.50 (1H, s)	12.43 (1H, s)	12.38 (1H, s)	12.43	12.38	12.36	12.31	12.44 (1H, s)	12.38	12.45	12.39
H6	6.19 (1H, d, *J* = 2.2 Hz)	6.20	6.21	6.20	6.22	6.21	6.23	6.20	6.22	6.21	6.23
7OH	10.78 (1H, br s)	10.80 (1H, s)	10.85 (1H, s)	10.82	10.87	10.85	10.91	10.81 (1H, s)	10.86	10.83	10.87
H8	6.38 (1H, d, *J* = 2.2 Hz)	6.42 (1H, d, *J* = 2.2 Hz)	6.38 (1H, d, *J* = 2.2 Hz)	6.43	6.39	6.49	6.45	6.42 (1H, d, *J* = 2.2 Hz)	6.39	6.43	6.40
H2′	7.25 (2H, s)	7.38 (1H, d, *J* = 2.2 Hz)	7.25	7.39	7.26	7.48	7.81 (2H, s)	7.37 (1H, d, *J* = 2.2 Hz)	7.26 (2H, s)	7.39	7.25 (2H, m)
3′OH	9.21 (2H, br s)	-	9.74	-	9.76 (2H, br s)	-	-	-	9.75 (2H, s)	-	9.77 (2H, s)
4′OH	8.80 (1H, br s)	9.83 (1H, s)	-	9.84	-	-	10.39 (1H, s)	9.86 (1H, s)	-	9.87	-
5′OH	9.21 (2H, br s)	9.52	9.74	9.53	9.76 (2H, br s)	10.34 (1H, s)	-	9.50 (1H, s)	9.75 (2H, s)	9.52	9.77 (2H, s)
H6′	7.25 (2H, s)	7.63 (1H, d, *J* = 2.2 Hz)	7.25	7.64	7.26	7.72	7.81 (2H, s)	7.64 (1H, d, *J* = 2.2 Hz)	7.26 (2H, s)	7.65	7.25 (2H, m)
3′O-H2″	-	2.63	-	2.64	-	2.62	2.68	2.60	-	2.60	-
3′O-H3″	-	1.15	-	1.17	-	1.14	1.17	1.65 (2H, m)	-	1.64 (1H, m)	-
3′O-H4″	-	-	-	-	-	-	-	1.29 (6H, m)	-	1.30 (4H, m)	-
3′O-H5″	-	-	-	-	-	-	-	1.29 (6H, m)	-	1.30 (4H, m)	-
3′O-H6″	-	-	-	-	-	-	-	1.29 (6H, m)	-	1.30 (4H, m)	-
3′O-H7″	-	-	-	-	-	-	-	1.39 (2H, dq, *J*	-	1.39 (1H, d, J =	-

								= 15, 7.5 Hz)		7.3 Hz)	
3′O-H8″	-	-	-	-	-	-	-	0.86 (3H, m)	-	0.88 (2H, br s)	-
4′O-H2″	-	-	2.60	-	2.60	2.62	-	-	2.57	-	2.57
4′O-H3″	-	-	1.13	-	1.14	1.14	-	-	1.65 (2H, m)	-	1.64 (2H, m)
4′O-H4″	-	-	-	-	-	-	-	-	1.29 (6H, m)	-	1.30 (6H, m)
4′O-H5″	-	-	-	-	-	-	-	-	1.29 (6H, m)	-	1.30 (6H, m)
4′O-H6″	-	-	-	-	-	-	-	-	1.29 (6H, m)	-	1.30 (6H, m)
4′O-H7″	-	-	-	-	-	-	-	-	1.38 (2H, dq, *J* = 15, 7.5 Hz)	-	1.39 (2H, d, *J* = 7.3 Hz)
4′O-H8″	-	-	-	-	-	-	-	-	0.88 (3H, m)	-	0.88 (3H, br s)
5′O-H2″	-	-	-	-	-	-	2.68	-	-	-	-
5′O-H3″	-	-	-	-	-	-	1.17	-	-	-	-

Myricetins acylated at [a] C3′-OH, [b] C4′-OH, [c] both C3′-OH and C4′-OH, or [d] both C3′-OH and C5′-OH.

Table S2. ^{13}C NMR (DMSO, 176 MHz) chemical shift (*δ*, ppm) of myricetin and acyl myricetins (MP$_1$, MP$_1$′, MP$_2$, MO$_1$, and MO$_1$′).

^{13}C location	M	MP$_1$		MP$_1$′		MP$_2$		MO$_1$		MO$_1$′	
		C3′ [a]	C4′ [b]	C3′	C4′	C3′,4′ [c]	C3′,5′ [d]	C3′	C4′	C3′	C4′
C2	146.76 (s)	145.56 (s)	145.41 (s)	145.56 (s)	145.41 (s)	144.09 (s)	144.39 (s)	145.58 (s)	145.44 (s)	145.58 (s)	145.44 (s)
C3	135.78 (s)	136.08 (s)	136.88 (s)	136.10 (s)	136.90 (s)	136.44 (s)	137.33 (s)	136.11 (s)	136.90 (s)	136.11 (s)	136.90 (s)
C4	175.67 (s)	175.77 (s)	176.07 (s)	175.78 (s)	176.08 (s)	175.86 (s)	176.12 (s)	175.80 (s)	176.08 (s)	175.80 (s)	176.08 (s)
C5	160.64 (s)	160.63 (s)	160.70 (s)	160.64 (s)	160.72 (s)	160.61 (s)	160.69 (s)	160.66 (s)	160.73 (s)	160.66 (s)	160.74 (s)
C6	98.06 (s)	98.14 (s)	98.20 (s)	98.16 (s)	98.22 (s)	98.23 (s)	98.29 (s)	98.16 (s)	98.22 (s)	98.17 (s)	98.23 (s)
C7	163.77 (s)	163.92 (s)	164.10 (s)	163.94 (s)	164.12 (s)	164.06 (s)	164.27 (s)	163.94 (s)	164.12 (s)	163.95 (s)	164.13 (s)
C8	93.10 (s)	93.30 (s)	93.17 (s)	93.32 (s)	93.19 (s)	93.58 (s)	93.43 (s)	93.30 (s)	93.18 (s)	93.31 (s)	93.19 (s)
C9	155.99 (s)	156.03 (s)	156.15 (s)	156.04 (s)	156.16 (s)	156.10 (s)	156.21 (s)	156.05 (s)	156.17 (s)	156.06 (s)	156.18 (s)
C10	102.88 (s)	102.94 (s)	103.04 (s)	102.95 (s)	103.06 (s)	103.01 (s)	103.13 (s)	102.96 (s)	103.07 (s)	102.96 (s)	103.07 (s)
C1′	120.70 (s)	120.76 (s)	128.14 (s)	120.78 (s)	128.16 (s)	121.06 (s)	128.64 (s)	120.79 (s)	128.14 (s)	120.80 (s)	128.14 (s)
C2′	107.08 (s)	113.56 (s)	106.73 (s)	113.58 (s)	106.74 (s)	112.65 (s)	119.88 (s)	113.54 (s)	106.73 (s)	113.55 (s)	106.74 (s)
C3′	145.62 (s)	138.73 (s)	150.00 (s)	138.74 (s)	150.02 (s)	143.31 (s)	139.43 (s)	138.76 (s)	150.04 (s)	138.76 (s)	150.05 (s)
C4′	135.78 (s)	139.77 (s)	128.11 (s)	139.78 (s)	128.11 (s)	131.74 (s)	143.34 (s)	139.80 (s)	128.14 (s)	139.80 (s)	128.14 (s)
C5′	145.62 (s)	146.34 (s)	150.00 (s)	146.36 (s)	150.02 (s)	150.28 (s)	139.43 (s)	146.35 (s)	150.04 (s)	146.35 (s)	150.05 (s)
C6′	107.08 (s)	112.14 (s)	106.73 (s)	112.14 (s)	106.74 (s)	113.26 (s)	119.88 (s)	112.16 (s)	106.73 (s)	112.17 (s)	106.74 (s)
3′O-C1″	-	171.94 (s)	-	171.96 (s)	-	171.45 (s)	172.08 (s)	171.17 (s)	-	171.18 (s)	-
3′O-C2″	-	26.55 (s)	-	26.57 (s)	-	26.38 (s)	26.59 (s)	33.15 (s)	-	33.15 (s)	-
3′O-C3″	-	8.78 (s)	-	8.80 (s)	-	8.81 (s)	8.71 (s)	24.29 (s)	-	24.29 (s)	-
3′O-C4″	-	-	-	-	-	-	-	28.29 (s)	-	28.29 (s)	-
3′O-C5″	-	-	-	-	-	-	-	28.22 (s)	-	28.23 (s)	-
3′O-C6″	-	-	-	-	-	-	-	31.08 (s)	-	31.06 (s)	-
3′O-C7″	-	-	-	-	-	-	-	21.96 (s)	-	21.96 (s)	-
3′O-C8″	-	-	-	-	-	-	-	13.85 (s)	-	13.86 (s)	-
4′O-C1″	-	-	171.45 (s)	-	171.48 (s)	170.76 (s)	-	-	170.67 (s)	-	170.69 (s)
4′O-C2″	-	-	26.45 (s)	-	26.46 (s)	26.52 (s)	-	-	33.09 (s)	-	33.09 (s)
4′O-C3″	-	-	8.85 (s)	-	8.87 (s)	8.88 (s)	-	-	24.38 (s)	-	24.39 (s)
4′O-	-	-	-	-	-	-	-	-	28.32	-	28.33

C4″									(s)		(s)
4′O-C5″	-	-	-	-	-	-	-	-	28.32 (s)	-	28.33 (s)
4′O-C6″	-	-	-	-	-	-	-	-	31.05 (s)	-	31.09 (s)
4′O-C7″	-	-	-	-	-	-	-	-	21.96 (s)	-	21.97 (s)
4′O-C8″	-	-	-	-	-	-	-	-	13.86 (s)	-	13.86 (s)
5′O-C1″	-	-	-	-	-	-	172.08 (s)	-	-	-	-
5′O-C2″	-	-	-	-	-	-	26.59 (s)	-	-	-	-
5′O-C3″	-	-	-	-	-	-	8.71 (s)	-	-	-	-

Myricetins acylated at [a] C3′-OH, [b] C4′-OH, [c] both C3′-OH and C4′-OH, or [d] both C3′-OH and C5′-OH.

Table S3. Compositions (%) of the peak fractions (MP_1, MP_1', MP_2, MO_1, and MO_1') and the mixtures (MP_1, MP_2, and MO_1) for the chemical stability, activity, and toxicity tests.

Fractions	Mixtures	Monoacylated [a]		Diacylated [b]	
		C3′ [c]	C4′ [d]	C3′,4′ [e]	C3′,5′ [f]
MP_1	-	37.5	62.5	-	-
MP_1'	-	41.2	58.8	-	-
MP_2	-	-	-	47.8	52.2
MO_1	-	41.7	58.3	-	-
MO_1'	-	39.2	60.8	-	-
-	MP_1	39.3	60.7	-	-
-	MP_2	-	-	47.8	52.2
-	MO_1	40.5	59.5	-	-

[a] Monoacylated or [b] diacylated myricetins. Myricetins acylated at [c] C3′-OH, [d] C4′-OH, [e] both C3′-OH and C4′-OH, or [f] both C3′-OH and C5′-OH.

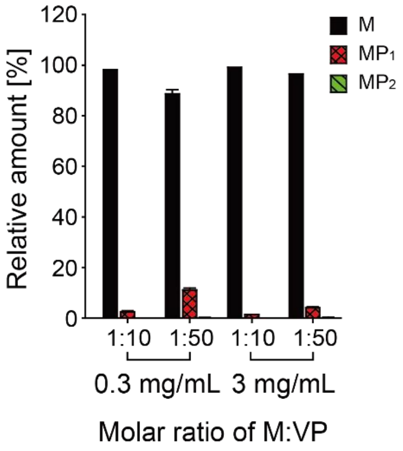

Fig. S1. Effect of concentration of myricetin and molar ratio of vinyl propionate on acylation efficiency in the absence of CaLB. Non-enzymatic propionylation was performed (myricetin concentration = 0.3 or 3 mg·mL^{-1}; molar ratio of myricetin-to-vinyl propionate = 1:10 or 1:50). M, myricetin; MP_1, monopropionyl-myricetin; MP_2, dipropionyl-myricetin.

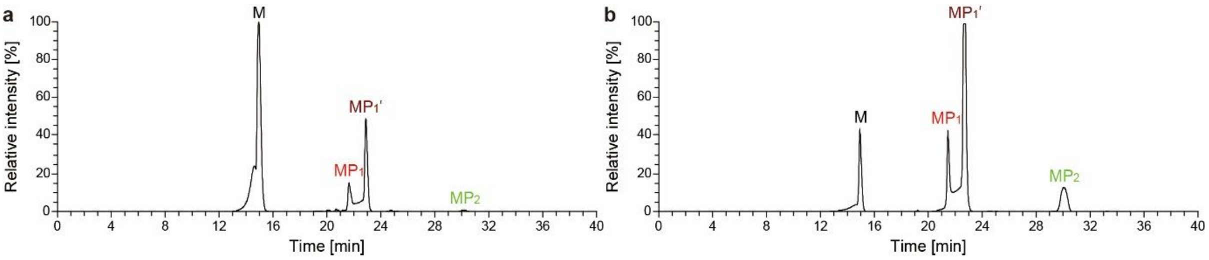

Fig. S2. Typical chromatograms of preparative high-performance liquid chromatography of the propionyl-myricetins synthesized (a) in the absence or (b) in the presence of CaLB for collecting the product fractions. M, myricetin; MP_1 and MP_1', monopropionyl-myricetin; MP_2, dipropionyl-myricetin.

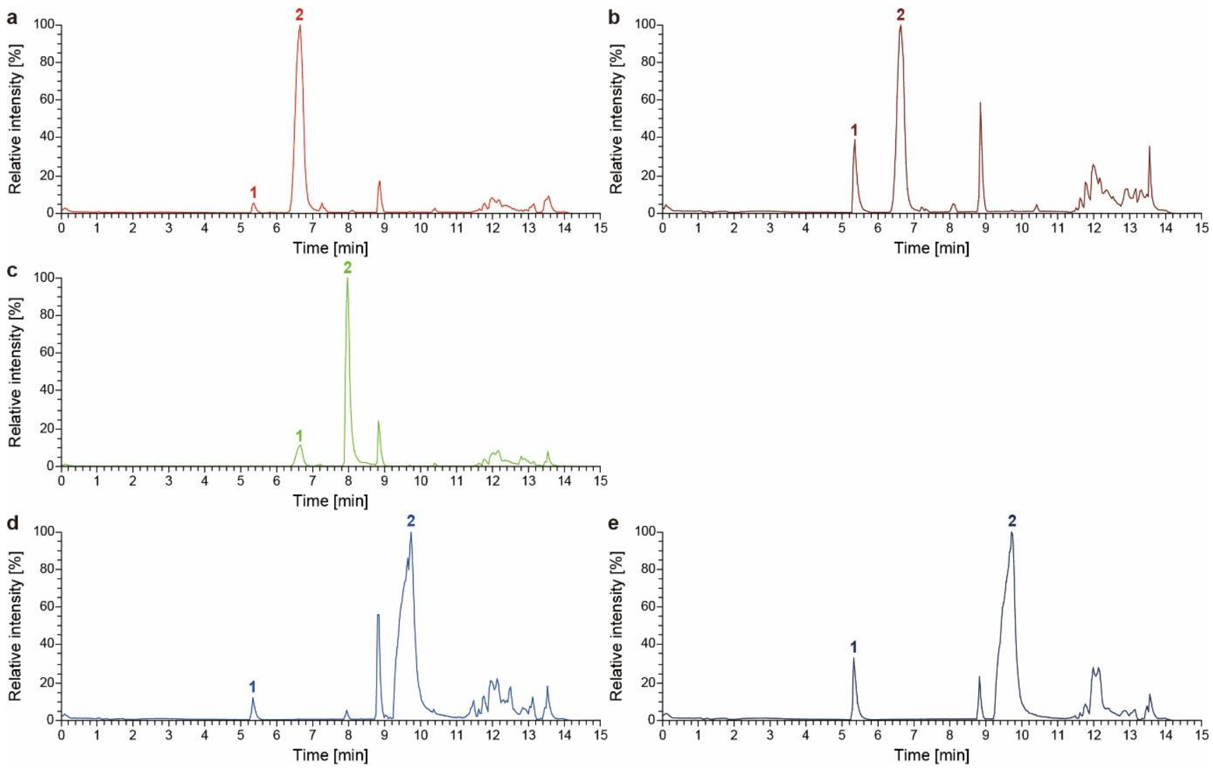

Fig. S3. Chromatograms of ultra-high performance liquid chromatography. Positively electrosprayed mass $[M+H]^+$ spectrometer chromatograms of the peak fractions; (a) MP_1, first peak fraction of MP_1 peaks; (b) MP_1', second peak fraction of MP_1 peaks; (c) MP_2, dipropionyl-myricetin; (d) MO_1, first peak fraction of MO_1 peaks; (e) MO_1', second peak fraction of MO_1 peaks.

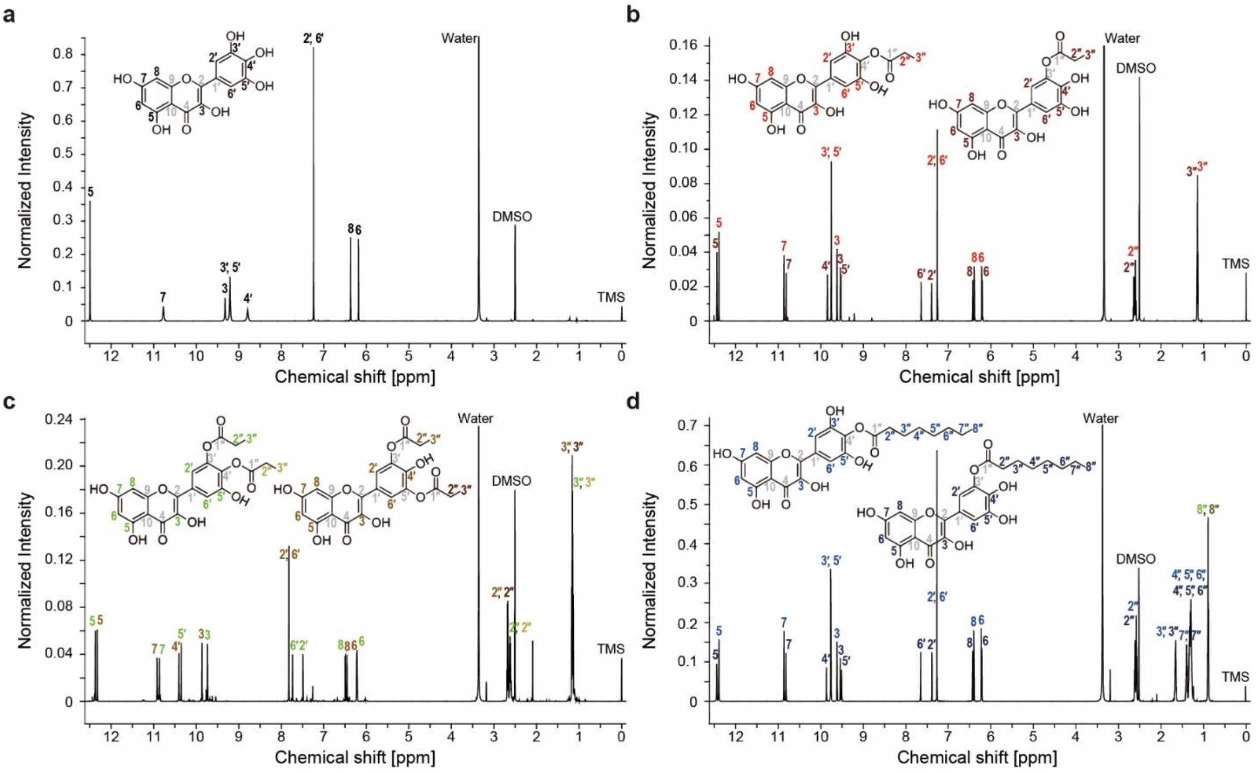

Fig. S4. ^{1}H NMR spectra of (a) myricetin and the peak fractions; (b) MP_1, (c) MP_2, and (d) MO_1. DMSO, dimethyl sulfoxide; TMS, tetramethylsilane.

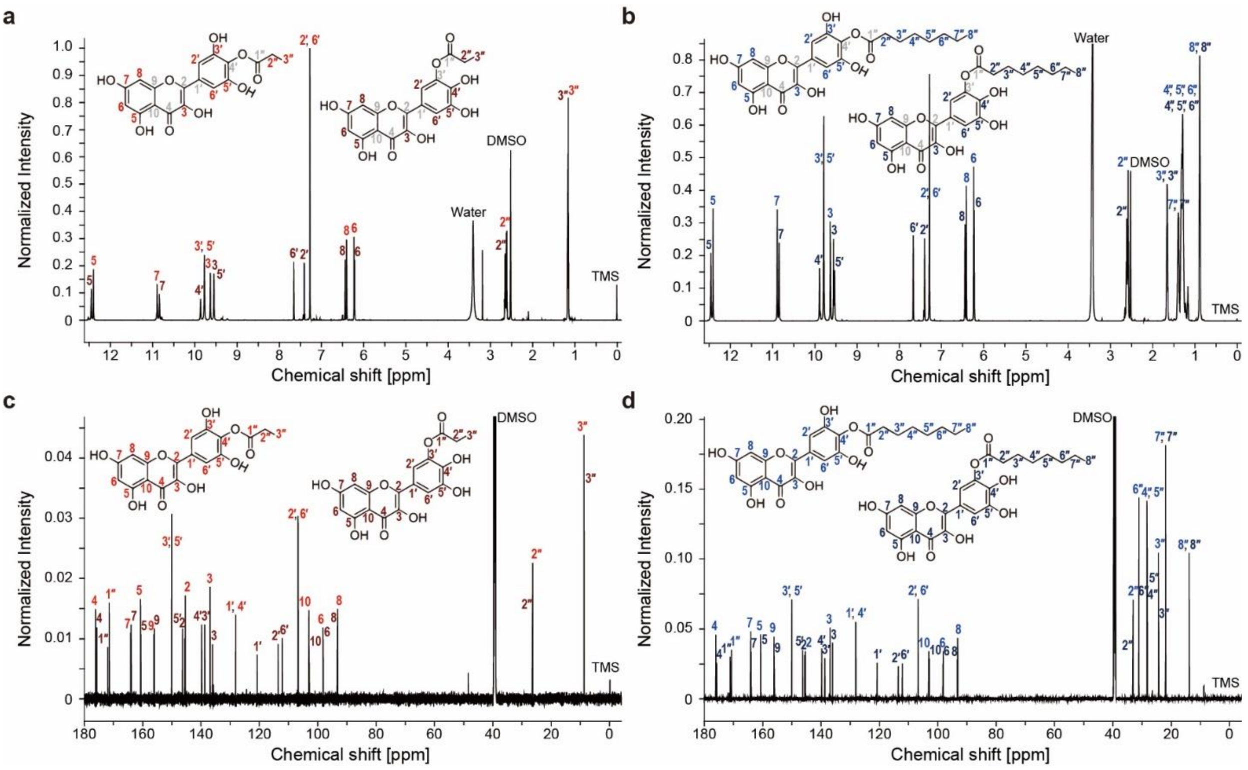

Fig. S5. (a, b) ^{1}H and (c, d) ^{13}C NMR spectra of the peak fractions; (a, c) MP$_1$′ and (b, d) MO$_1$′. DMSO, dimethyl sulfoxide; TMS, tetramethylsilane.

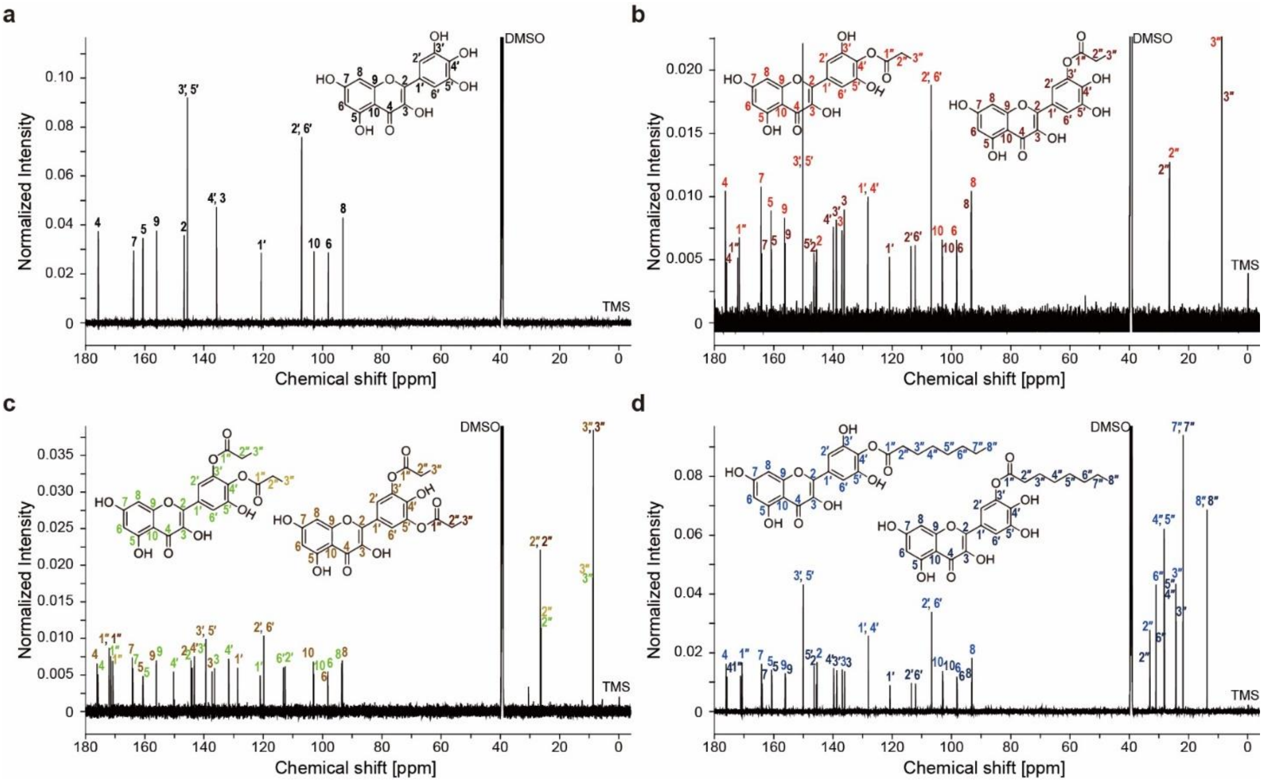

Fig. S6. ^{13}C NMR spectra of (a) myricetin and the peak fractions; (b) MP_1, (c) MP_2, and (d) MO_1. DMSO, dimethyl sulfoxide; TMS, tetramethylsilane.

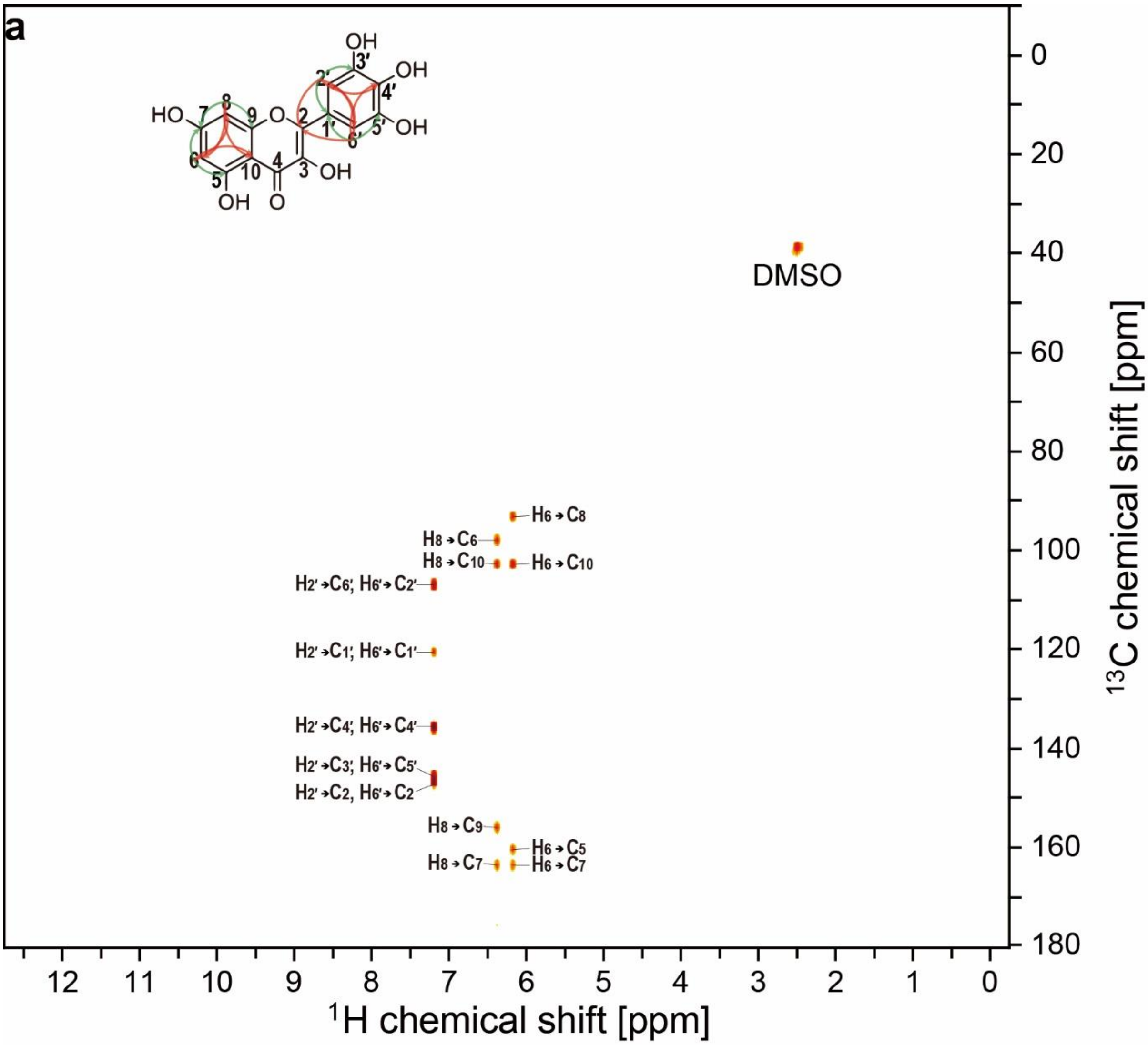

a
HO
OH
OH
OH
OH
OH
O
O
DMSO
H8→C6
H8→C10
H6→C8
H6→C10
H2'→C6'; H6'→C2'
H2'→C1'; H6'→C1'
H2'→C4'; H6'→C4'
H2'→C3'; H6'→C5'
H2'→C2, H6'→C2
H8→C9
H8→C7
H6→C5
H6→C7
^{1}H chemical shift [ppm]
^{13}C chemical shift [ppm]

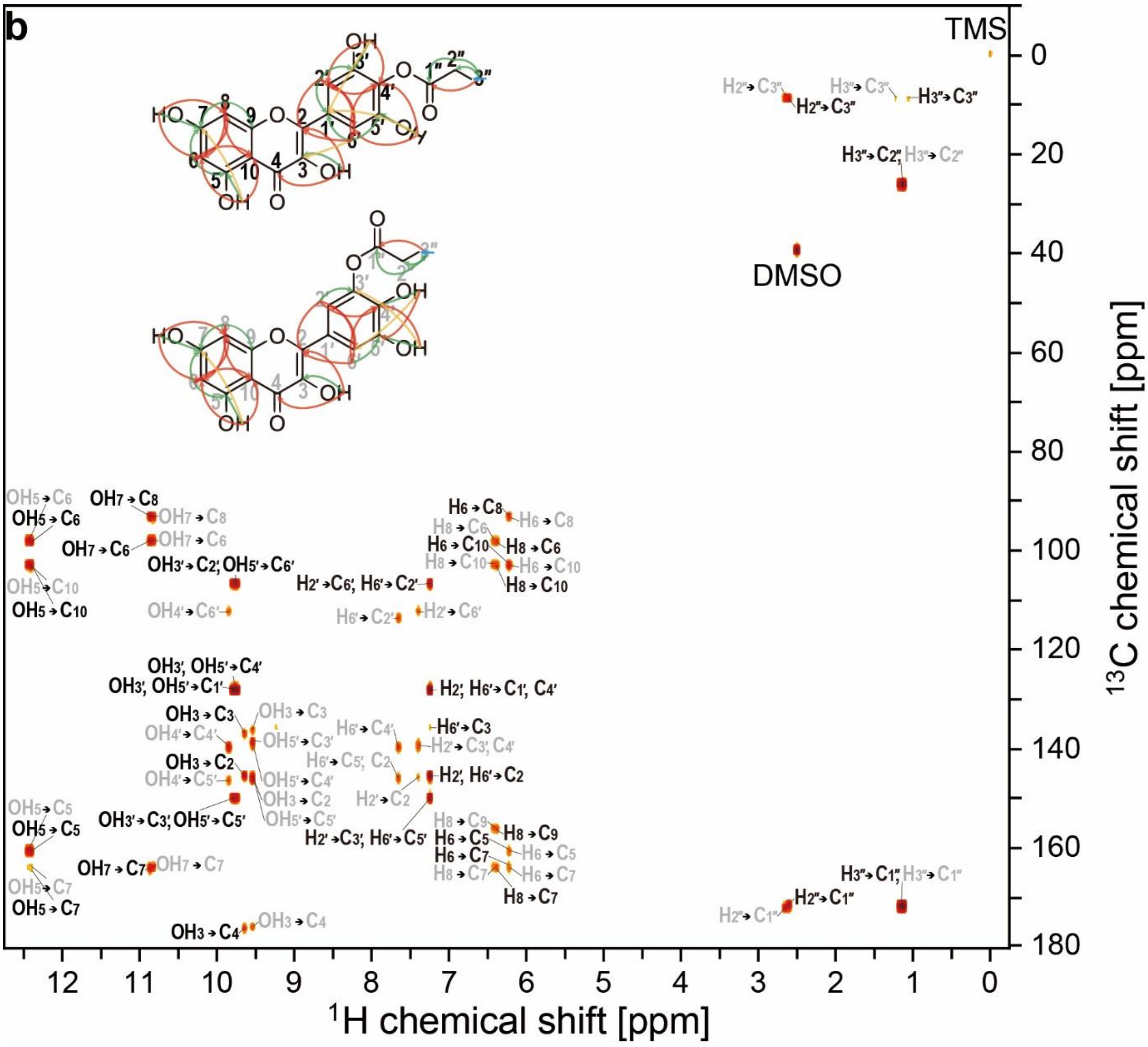
b
TMS
DMSO
H2″→C3″
H3″→C3″
H3″→C2″
OH5→C6
OH7→C8
OH7→C6
OH5→C10
OH3′→C2′, OH5′→C6′
OH4′→C6′
H6→C8
H8→C6
H6→C10
H8→C10
H2′→C6′, H6′→C2′
H6′→C2′
H2′→C6′
OH3′, OH5′→C4′
OH3′, OH5′→C1′
H2′, H6′→C1′, C4′
OH3→C3
OH4′→C4′
OH5′→C3′
H6′→C4′
H6′→C3
H2′→C3′, C4′
OH3→C2
OH4′→C5′
OH5′→C4′
H6′→C5′, C2
H2′→C2
H2′, H6′→C2
OH3′→C3′, OH5′→C5′
OH5′→C5′
H2′→C3′, H6′→C5′
H8→C9
H6→C5
H6→C7
H8→C7
OH5→C5
OH5→C7
OH7→C7
H3″→C1″
H2″→C1″
OH3→C4
¹H chemical shift [ppm]
¹³C chemical shift [ppm]

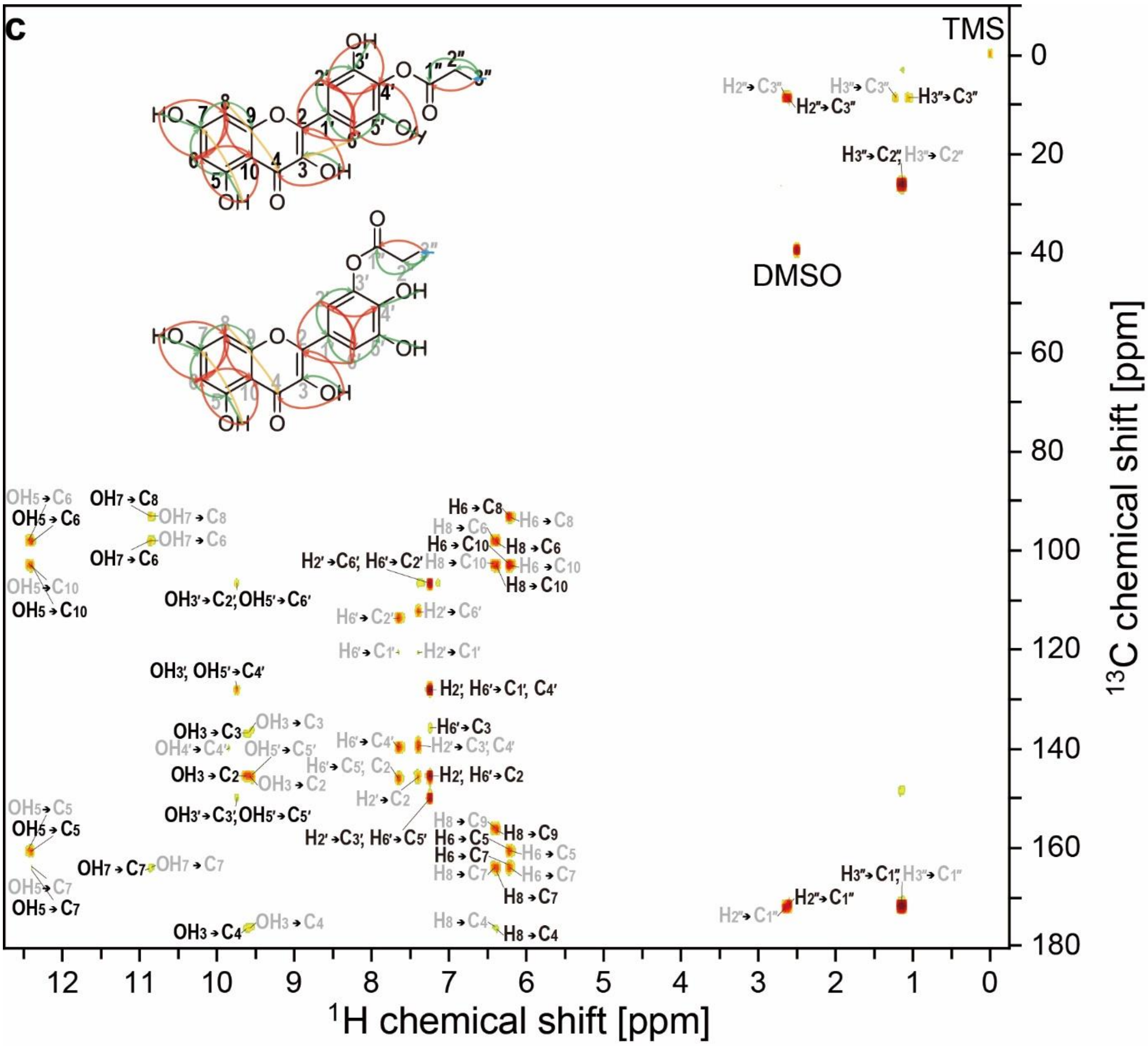

c
TMS
DMSO
H2″→C3″
H3″→C3″
H3″→C2″
H2″→C1″
H3″→C1″
OH5→C6
OH5→C10
OH7→C8
OH7→C6
OH3′→C2′; OH5′→C6′
OH3′; OH5′→C4′
OH3→C3
OH3→C2
OH3′→C3′; OH5′→C5′
OH5→C5
OH5→C7
OH7→C7
OH3→C4
H6→C8
H6→C10
H8→C6
H8→C10
H2′→C6′; H6′→C2′
H2′; H6′→C1′, C4′
H6′→C3
H2′; H6′→C2
H2′→C3′; H6′→C5′
H8→C9
H6→C5
H6→C7
H8→C7
H8→C4
^{1}H chemical shift [ppm]
^{13}C chemical shift [ppm]

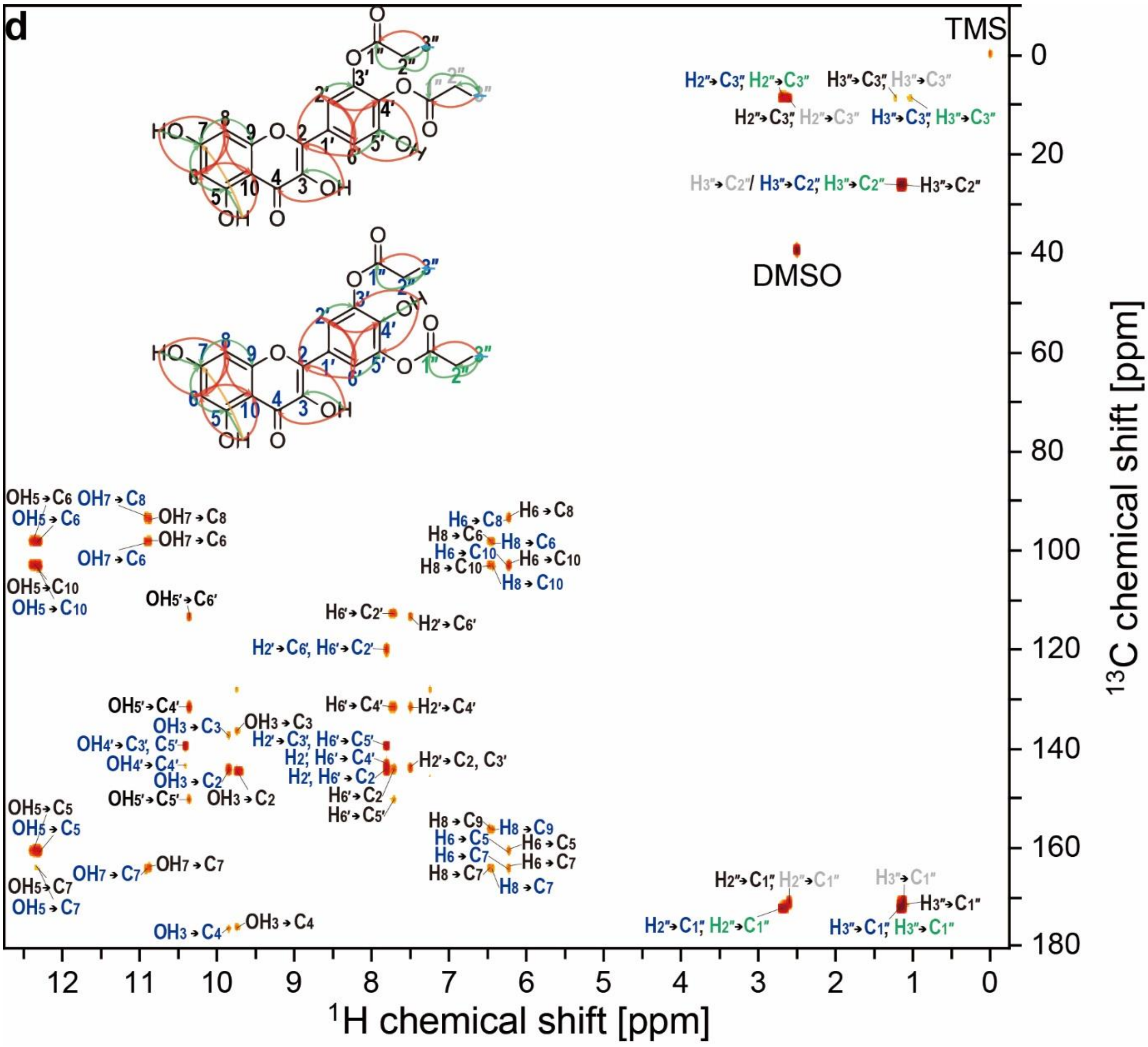

Fig. S7. ^{1}H-^{13}C heteronuclear multiple bond correlation (HMBC)-NMR spectra of (a) myricetin and the peak fractions; (b) MP_1, (c) MP_1', and (d) MP2. DMSO, dimethyl sulfoxide; TMS, tetramethylsilane.

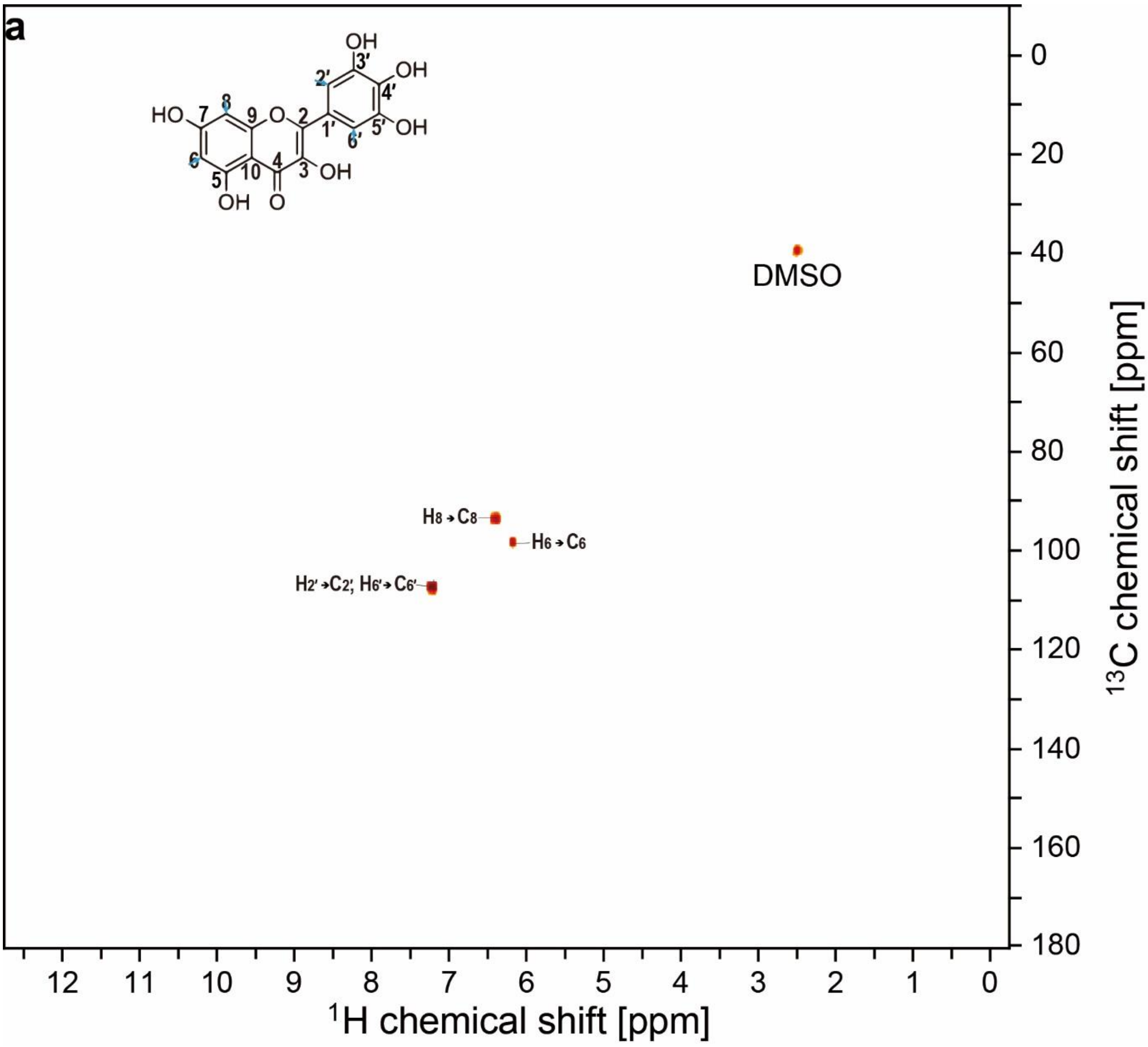
a
HO
OH
OH
OH
OH
O
O
DMSO
H8 → C8
H6 → C6
H2′ → C2′; H6′ → C6′
¹H chemical shift [ppm]
¹³C chemical shift [ppm]

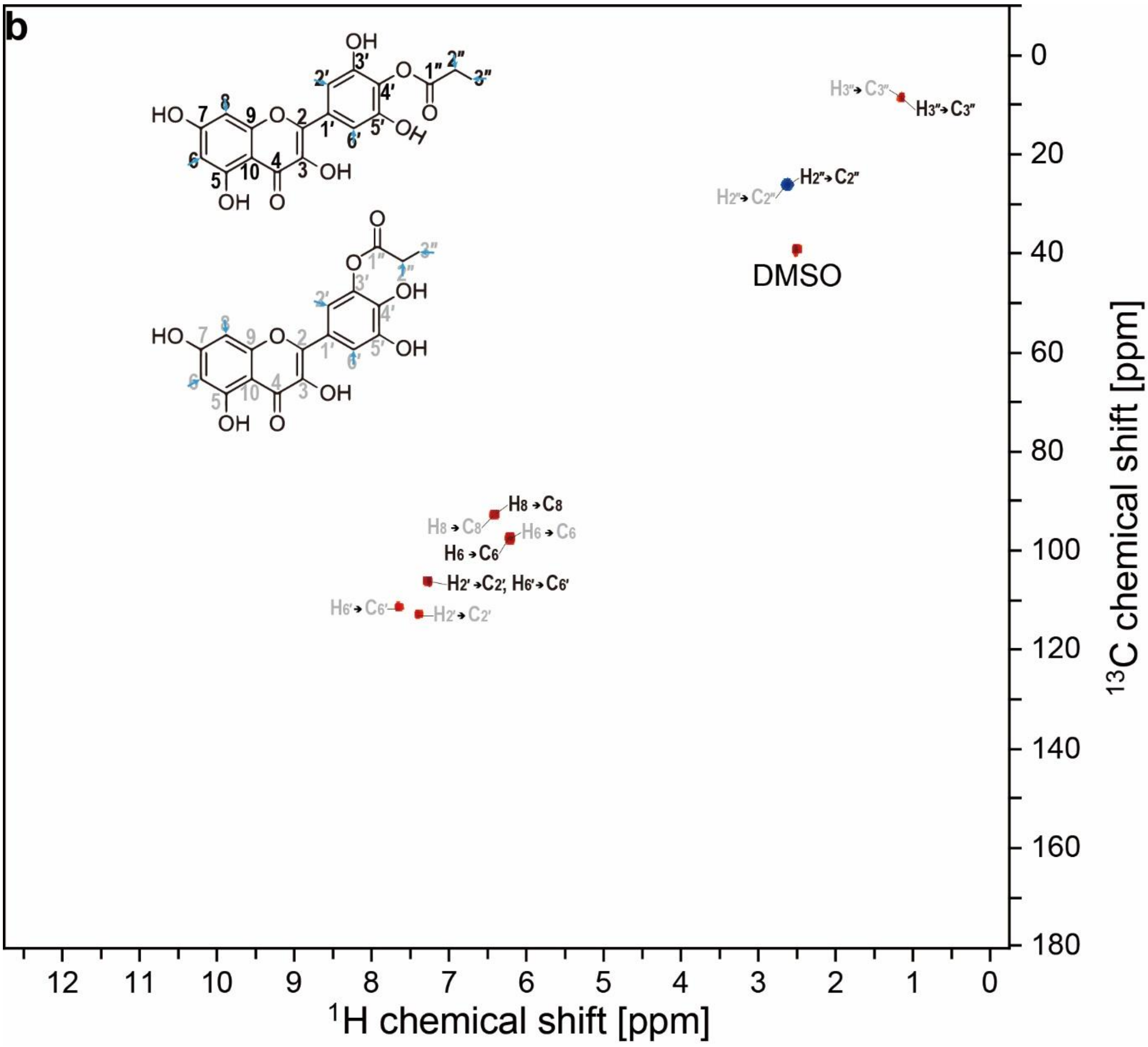
b
DMSO
H3″→C3″
H3″→C3″
H2″→C2″
H2″→C2″
H8→C8
H8→C8
H6→C6
H6→C6
H2′→C2′; H6′→C6′
H6′→C6′
H2′→C2′
^{1}H chemical shift [ppm]
^{13}C chemical shift [ppm]

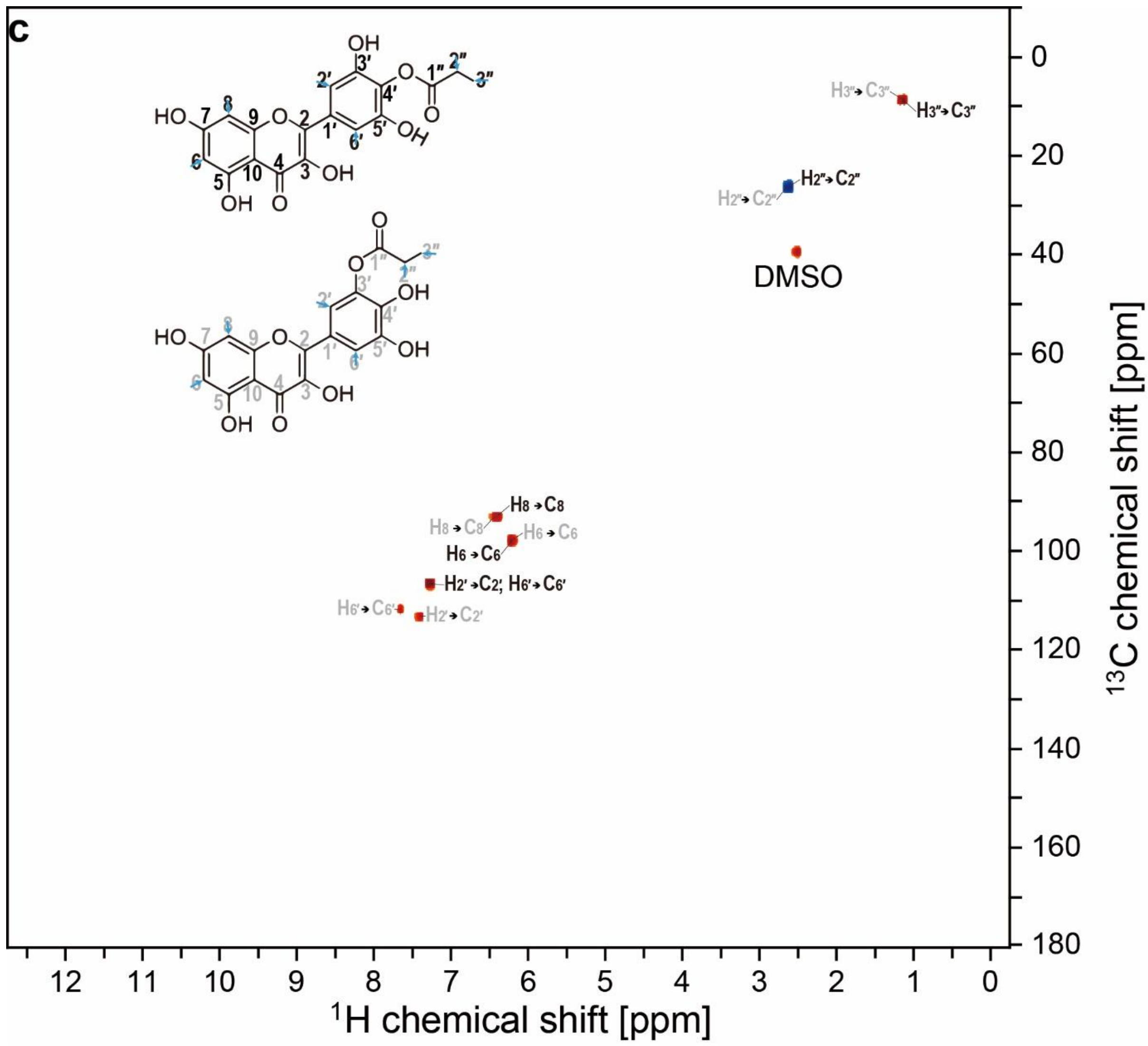
c
DMSO
H3″→C3″
H2″→C2″
H8→C8
H6→C6
H2′→C2′; H6′→C6′
H6′→C6′
H2′→C2′
¹H chemical shift [ppm]
¹³C chemical shift [ppm]

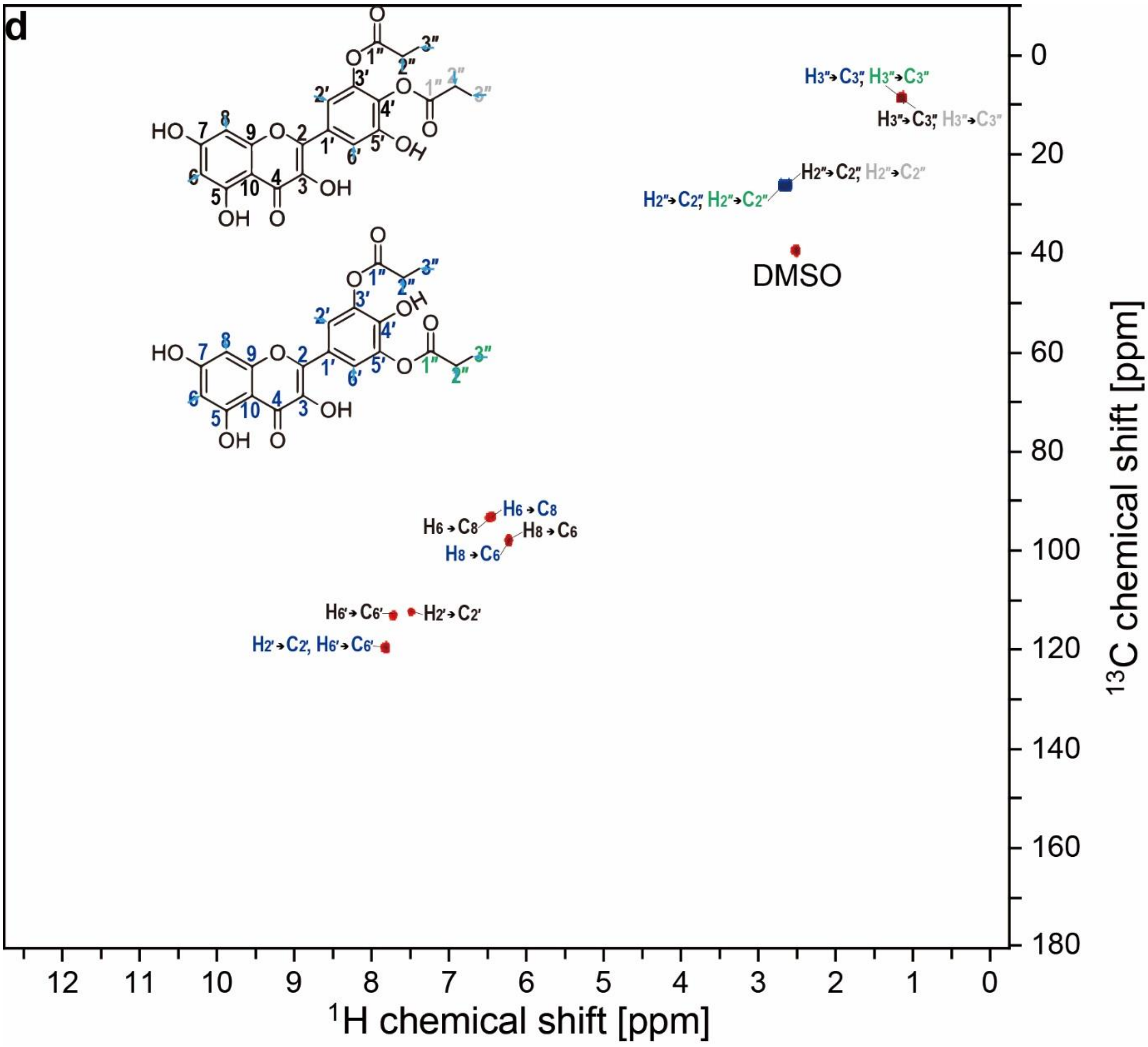

Fig. S8. ^{1}H-^{13}C heteronuclear single quantum coherence correlation (HSQC)-NMR spectra of (a) myricetin and the peak fractions; (b) MP1, (c) MP1′, and (d) MP2. DMSO, dimethyl sulfoxide.

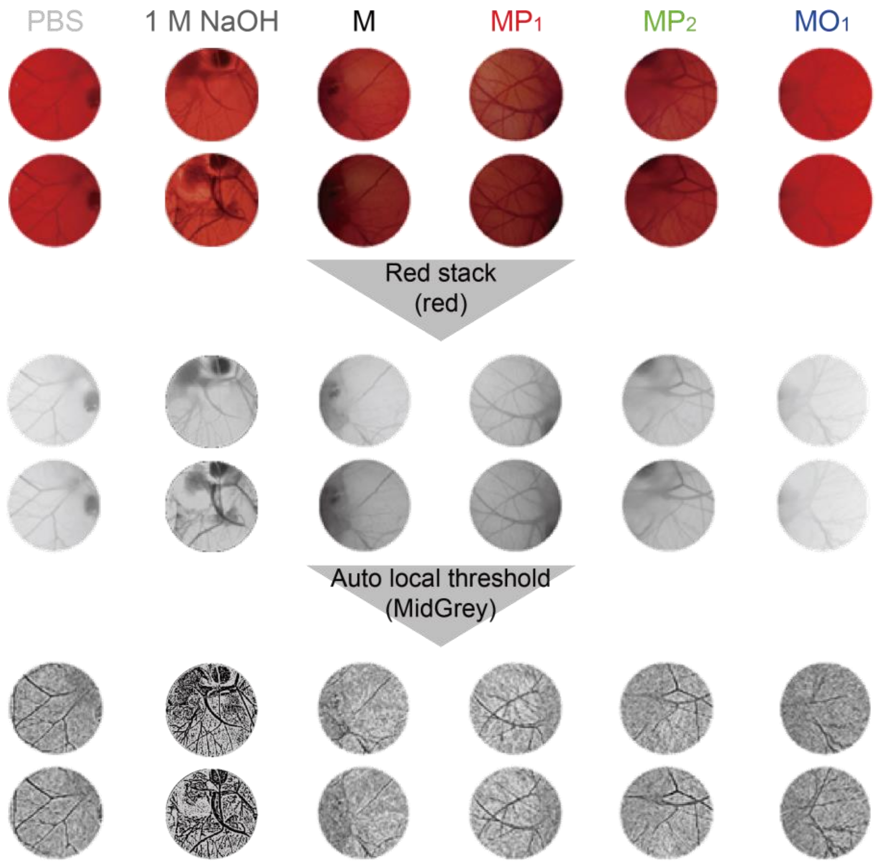

Fig. S9. HET-CAM tests of acyl myricetins. 2 mL of test solutions including M, MP_1, MP_2, MO_1 mixtures were placed on the CAM. A mixture of PBS (1.9 mL) and DMSO (100 μL) (indicated as 'PBS'), and 1 M NaOH aqueous solution were used as negative and positive controls, respectively. The CAM images were analyzed using ImageJ (http://rsb.info.nih.gov/ij/); loaded individually into ImageJ, cropped to a fixed size to exclude the shell, and converted to grayscale. The CAM images, grayscaled with red stack, were adjusted using the MidGrey method, and the area fraction (%Area) values of black pixels in the adjusted CAM images were measured and recorded.

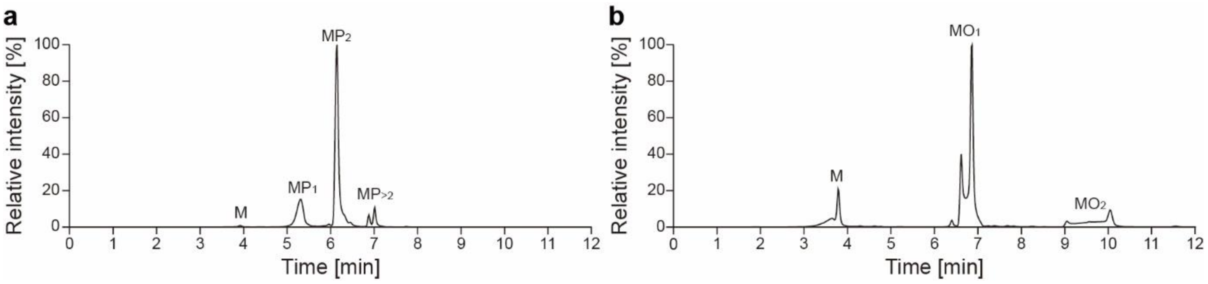

Fig. S10. Typical chromatograms of high-performance liquid chromatography of (a) the propionyl- and (b) octanoyl-myricetins. MP_1, monopropionyl-myricetin; MP_2, dipropionyl-myricetin; $MP_{>2}$, multipropionyl-myricetin; MO_1, monooctanoyl-myricetin; MO_2, dioctanoyl-myricetin.
